# Supplementary material for: High-throughput 3D spheroid screens identify microRNA sensitizers for improved thermoradiotherapy in locally advanced cancers
Source: Mol Ther Nucleic Acids. 2025 Mar 5;36(2):102500. doi: 10.1016/j.omtn.2025.102500 (PMC11979520; doi:10.1016/j.omtn.2025.102500)
Supplement: Document S1. Figures S1–S5 [file mmc1.pdf]

OMTN, Volume *36*

Supplemental information

High-throughput 3D spheroid screens identify microRNA sensitizers for improved thermoradiotherapy in locally advanced cancers

MengFei Xu, Mark A. van de Wiel, Dominika Martinovičová, Angelina Huseinovic, Victor W. van Beusechem, Lukas J.A. Stalpers, Arlene L. Oei, Renske D.M. Steenbergen, and Barbara C. Snoek

Table S1: Overview of miRNAs selected for the discovery screen, validation screen and subsequent functional validation, including obtained relative effect sizes. FDR, false discovery rate.

Table S2: Overview of predicted miRNA targets associated with DNA damage response, excluding targets associated with DNA repair pathways HR and NHEJ. Predicted targets for each miRNA are marked with X. miRNAs with no predicted targets— miR-27a, miR-92a, miR-106b, and miR-3158— are not included in the table.

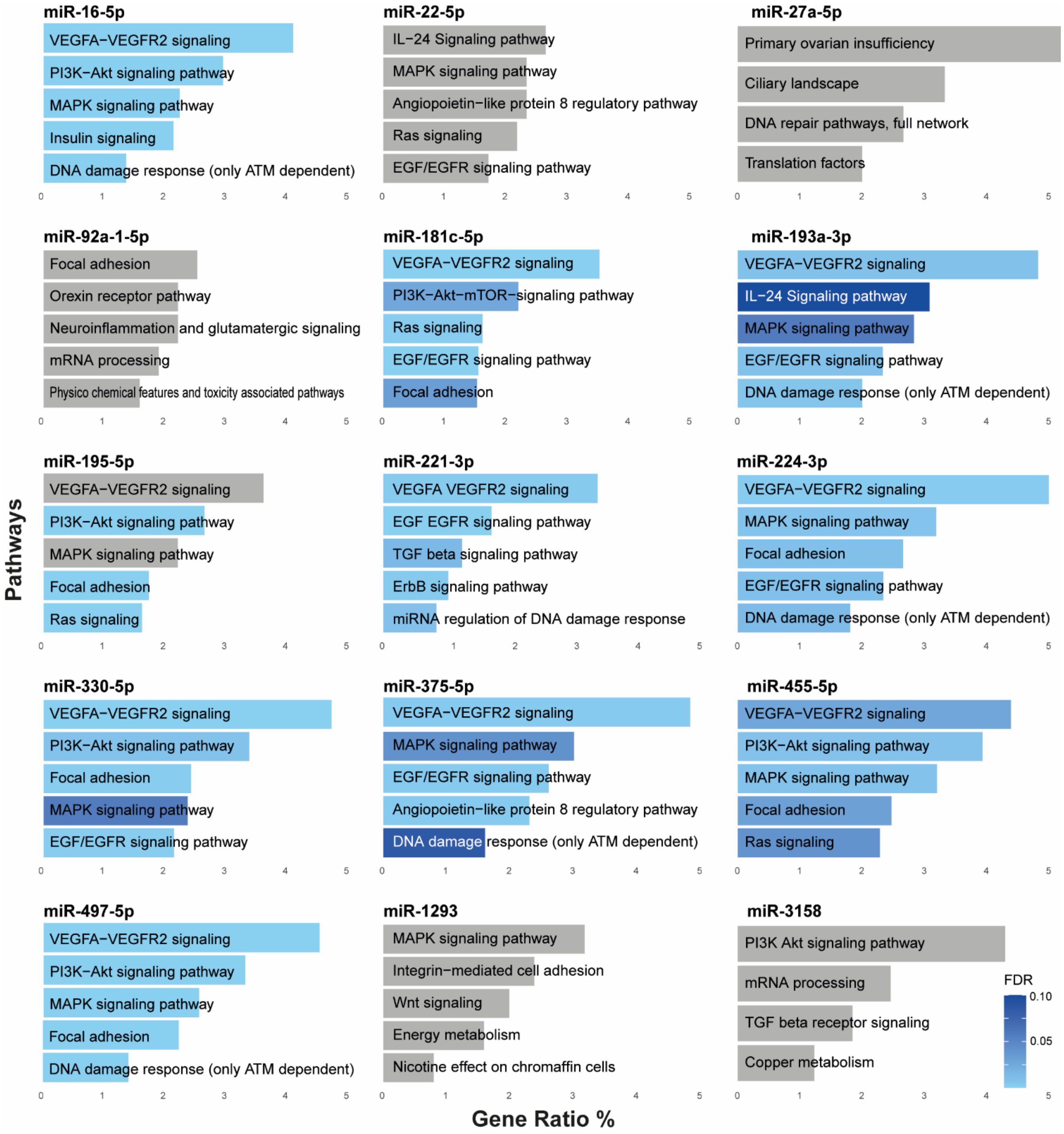

Figure S1: Overview of significantly enriched pathways for each miRNA. The top five enriched pathways or all pathways (in case less than five) were shown. Gene ratio indicates the percentage of genes over the total genes in the given pathway. Insignificant enrichments are indicated in gray. FDR, false discovery rate.

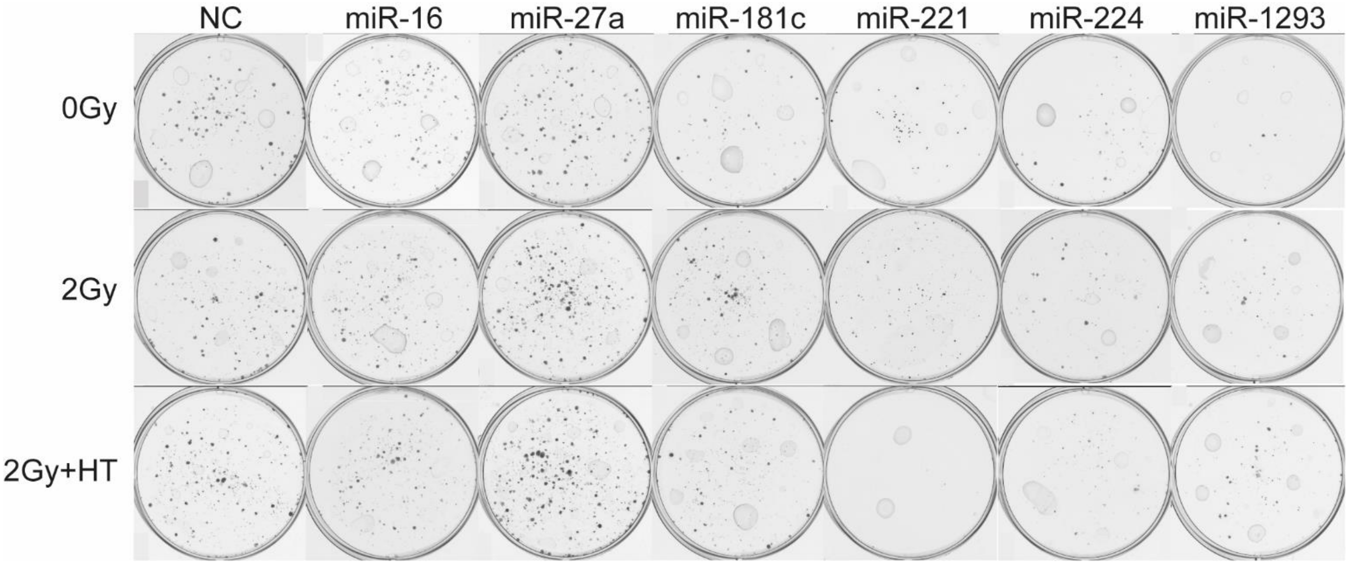

Figure S2: Clonogenic assays of different treatment conditions combined with miRNA transfection. Representative images of clonogenic assays for each miRNA are shown with non-treatment (0Gy), irradiation (2 Gy), thermoradiotherapy (2 Gy+HT) in SiHa cells.

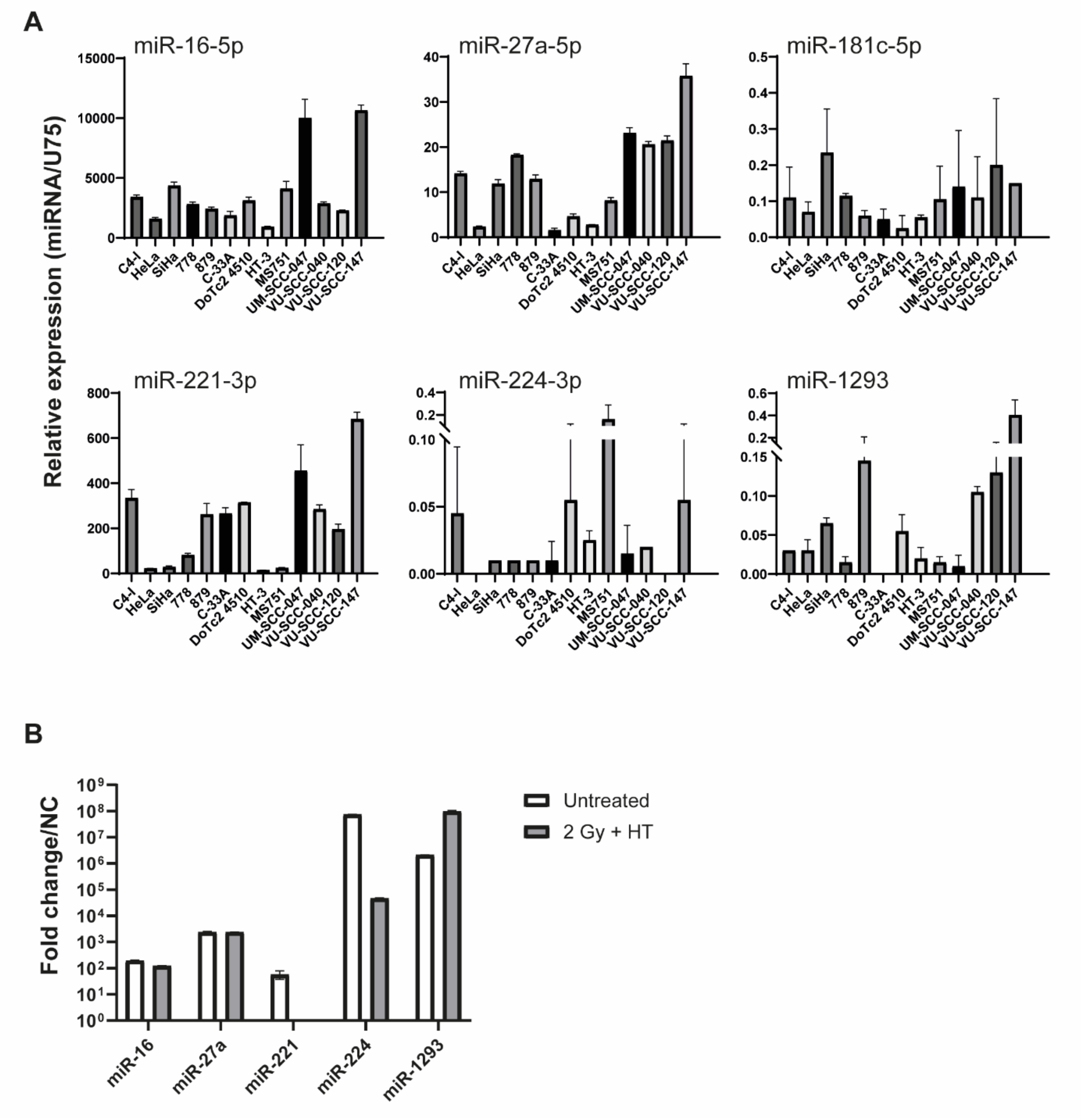

Figure S3: miRNA expression levels quantified by RT-qPCR. A: The expression level of miR-16-5p, miR-27a-5p, miR-181c-5p, miR-221-3p, miR-224-3p, and miR-1293 were quantified in relation to reference gene U75 across cancer cell lines included in the high-throughput miRNA screens. **B**: relative miRNA expression fold change compared to NC (fold change = 1) in SiHa cells 48 hours post-treatment are indicated. Untreated refers to miRNA transfected cells only, while 2 Gy+HT refers to cells that received thermoradiotherapy combined with miRNA transfection. For miR-221, only untreated cells were included. The mean ± SD are shown.

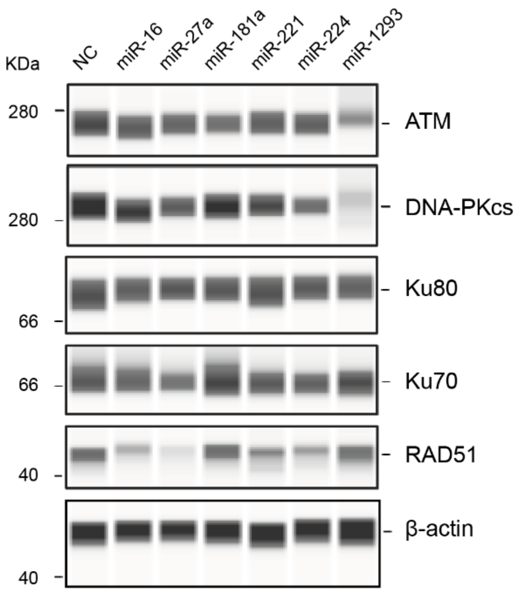

Figure S4: Western Blot analysis of predicted miRNA targets associated with DNA repair pathways. Whole cell extracts from SiHa cells collected 24 hours after miRNA mimic transfection were analyzed for ATM, DNA-PKcs, Ku80, Ku70, and RAD51 expression using Simple Western. β-actin was used as a loading control. The bands shown are digitally generated representations derived from light signal peaks of the electropherograms produced by Simple Western. Data from one of two independent experiments are shown. NC, negative control.

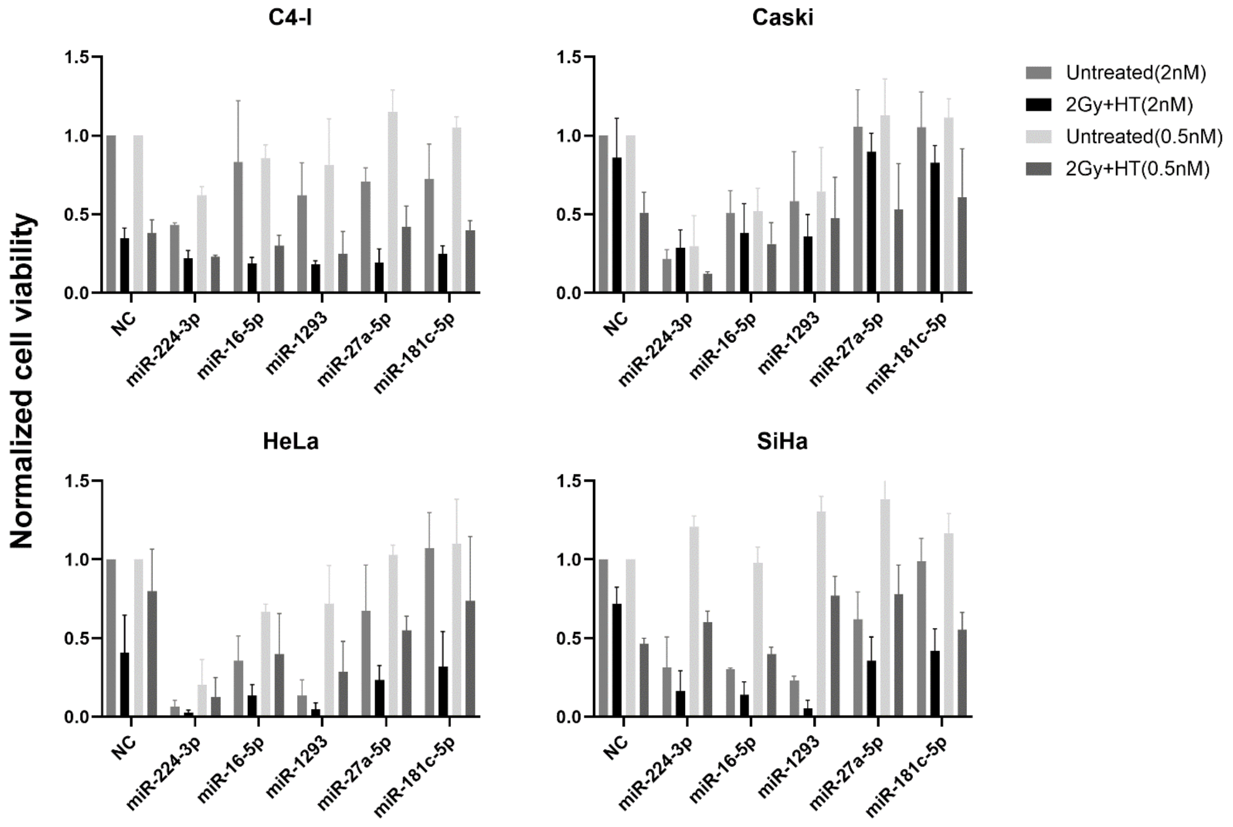

Figure S5: Cell viability results for the top five significant miRNAs from the validation screen at different miRNA mimic concentrations. Viability is normalized to the negative control (NC) for each cell line (C4-I, CaSki, HeLa, and SiHa) at each miRNA mimic concentration (2 nM or 0.5 nM). Data represent mean ± SD. Untreated refers to miRNA transfected cells only, while 2 Gy+HT refers to cells that received thermoradiotherapy combined with miRNA transfection.
