# Supplementary material for: High-throughput 3D spheroid screens identify microRNA sensitizers for improved thermoradiotherapy in locally advanced cancers
Source: Mol Ther Nucleic Acids. 2025 Mar 5;36(2):102500. doi: 10.1016/j.omtn.2025.102500 (PMC11979520; doi:10.1016/j.omtn.2025.102500)
Supplement: Document S2. Article plus supplemental information [file mmc4.pdf]

Molecular Therapy
Nucleic Acids
Original Article

High-throughput 3D spheroid screens identify microRNA sensitizers for improved thermoradiotherapy in locally advanced cancers

MengFei Xu,[1,2] Mark A. van de Wiel,[3] Dominika Martinovičová,[1,2] Angelina Huseinovic,[1,2] Victor W. van Beusechem,[4,5,6] Lukas J.A. Stalpers,[7] Arlene L. Oei,[5,7,8] Renske D.M. Steenbergen,[1,2] and Barbara C. Snoek[5,7,8]

[1]Amsterdam UMC, Vrije Universiteit Amsterdam, Pathology, De Boelelaan 1117, 1081 HV Amsterdam, the Netherlands; [2]Cancer Center Amsterdam, Imaging and Biomarkers, De Boelelaan 1117, 1081 HV Amsterdam, the Netherlands; [3]Amsterdam UMC, Vrije Universiteit Amsterdam, Epidemiology and Data Science, De Boelelaan 1117, 1081 HV Amsterdam, the Netherlands; [4]Amsterdam UMC, Vrije Universiteit Amsterdam, Medical Oncology, De Boelelaan 1117, 1081 HV Amsterdam, the Netherlands; [5]Cancer Center Amsterdam, Cancer Biology and Immunology, De Boelelaan 1117, 1081 HV Amsterdam, the Netherlands; [6]Amsterdam Infection and Immunity Institute, Cancer Immunology, De Boelelaan 1117, 1081 HV Amsterdam, the Netherlands; [7]Amsterdam UMC, University of Amsterdam, Radiation Oncology, Meibergdreef 9, 1105 AZ Amsterdam, the Netherlands; [8]Laboratory for Experimental Oncology and Radiobiology, Amsterdam UMC, University of Amsterdam, Meibergdreef 9, 1105 AZ Amsterdam, the Netherlands

Chemoradiotherapy is the standard of care for many locally advanced cancers, including cervical and head and neck cancers, but many patients cannot tolerate chemotherapy. Clinical trials have shown that radiotherapy combined with hyperthermia (thermoradiotherapy) may be equally effective, yet it yields a suboptimal overall survival of patients, emphasizing the need for improvement. MicroRNAs (miRNAs), short non-coding RNA sequences, are often dysregulated in cancer and exhibit significant potential as radiosensitizers by targeting genes associated with the DNA damage response. In this study, high-throughput miRNA screening of four cervical cancer cell lines identified 55 miRNAs with significant sensitizing potential, with 18 validated across 10 additional cancer cell lines (6 cervical and 4 head and neck). Functional studies of 6 miRNAs, including miR-16, miR-27a, miR-181c, miR-221, miR-224, and miR-1293, showed that they reduced DNA damage repair by downregulating ATM, DNA-PKcs, Ku70/80, and RAD51. Additionally, differential expression of miR-27a, miR-221, and miR-224 in treatment-sensitive versus treatment-resistant patients indicated their predictive biomarker potential for treatment response of cervical cancer patients. Conclusively, this study has identified 18 promising miRNAs for the development of sensitizers for thermoradiotherapy and may provide potential biomarkers for predicting treatment response in locally advanced cancers.

INTRODUCTION

Chemoradiotherapy (radiation combined with chemotherapy) is the standard treatment for locally advanced cervical cancer and head and neck cancers. However, many patients cannot tolerate chemotherapy because of frailty and side effects (neurotoxicity, nephrotoxicity, bone marrow depression); thermoradiotherapy (radiation therapy combined with hyperthermia) serves as a well-established alternative treatment strategy.[1] Preclinical and clinical studies have demonstrated a clear benefit of thermoradiotherapy over radiotherapy alone, and non-inferior to chemoradiotherapy with reduced toxicity.[2–5]

The clinical use of hyperthermia as a sensitizer of radiotherapy or chemotherapy is increasingly gaining recognition. Currently, thermoradiotherapy is applied for the treatment of recurrent breast cancer and advanced cervical cancer, and numerous studies have demonstrated the clinical efficacy of thermoradiotherapy for treatment of bladder cancer, esophageal cancer, head and neck cancers, and melanoma.[1,6,7]

Despite these encouraging findings, the clinical outcome of patients with locally advanced cancer remains poor. For locally advanced head and neck cancer patients, the 5-year overall survival rate ranges between 50% and 78%, depending on the specific tumor site.[8] The 5-year overall survival rate for locally advanced cervical cancer patients treated with thermoradiotherapy ranges between 40% and 60%.[9–11] Bladder cancer patients treated with thermoradiotherapy show a reported 3-year overall survival rate of only 28%.[3] These observations underscore the clinical need for improving treatment outcomes.

Hyperthermia is employed as a sensitizer of radiotherapy. During hyperthermia, the tumor is locally heated to mild temperatures ranging between 39°C and 42°C for approximately 1 h. This process has been

Received 23 August 2024; accepted 28 February 2025;
https://doi.org/10.1016/j.omtn.2025.102500.

Correspondence: Barbara C. Snoek, van der Boechorststraat 6B, 1081 BT Amsterdam, the Netherlands.
E-mail: b.snoek2@amsterdamumc.nl

Published by Elsevier Inc. on behalf of The American Society of Gene and Cell Therapy.
This is an open access article under the CC BY license (http://creativecommons.org/licenses/by/4.0/).

demonstrated to enhance cell sensitivity to radiation through various mechanisms, such as (1) inhibition of radiation-induced DNA damage repair due to temporarily inhibiting the homologous recombination (HR) DNA damage repair pathway, (2) sensitization of S phase cells, and (3) targeting tumor cells in hypoxic and nutrient-deprived areas.[1,12,13] In addition to its radiosensitizing properties, hyperthermia has been found to trigger systemic anti-tumor immune responses.[14–16]

Previously, we have demonstrated that the high specificity of hyperthermia against human papillomavirus (HPV)$^+$ cervical cancers results from its ability to temporarily restore p53 function by disrupting the binding between the HPV oncoprotein E6 and p53.[17] This p53 restoration leads to induced tumor cell apoptosis following sublethal DNA damage caused by irradiation. In addition, Wang et al. found that hyperthermia leads to the downregulation of HPV oncoprotein E7, effectively reversing HPV-associated carcinogenesis *in vitro* and *in vivo*.[18] Furthermore, Jiang et al. showed that phosphorylation of phospholipid scramblase 3, a protein often associated with cancer, is involved in the signal transduction mechanism underlying hyperthermia-induced apoptosis in tongue squamous cell carcinoma.[19]

MicroRNAs (miRNAs) are a class of small non-coding RNAs approximately 20–25 nt in length, which play a pivotal role in post-transcriptional gene regulation and are often dysregulated in cancer.[20,21] miRNAs have been implicated in tumor radiosensitivity by targeting key components of the DNA damage response.[22] However, most studies have focused on individual miRNAs. No studies have investigated the effects of miRNA modulation on thermoradiotherapy.

In the present study, we aim to discover miRNAs that are modifiers of thermoradiotherapy and are potential agents for the development of sensitizers of thermoradiotherapy. Here, we use a high-throughput miRNA screen in three-dimensional (3D) tumor cell spheroids, a model that we recently developed and further optimized to assess the effectiveness of thermoradiotherapy.[23] Comprehensive target prediction, combining computational and experimental approaches, were used to investigate the association of identified miRNAs with DNA damage repair. The function of miRNAs was subsequently studied with clonogenic assays, γ-H2AX foci staining, and automated western blots. The biomarker potential was assessed using miRNA expression profiles of treatment-sensitive and treatment-resistant patients obtained from The Cancer Genome Atlas (TCGA) cervical cancer cohort.[24]

RESULTS

Discovery screen identifies 55 miRNAs as potent sensitizers for thermoradiotherapy

To identify potential sensitizers for thermoradiotherapy, we developed a high-throughput miRNA screening method using 3D spheroid models and cell viability as readout. The discovery screen entailed transfection of cervical cancer cells with 378 miRNA mimics (see materials and methods for selection criteria and Table S1) in 384-well plates followed by irradiation at 2 Gy and hyperthermia at 42°C for 60 min. Three days post-treatment, the cells were assayed for viability. The strategy of this study is outlined in Figure 1A.

Given that thermoradiotherapy is clinically applied for the treatment of locally advanced cervical cancer, we conducted our discovery screen across four cervical cancer cell lines (C-4 I, CaSki, HeLa, and SiHa). We performed a comprehensive analysis of the obtained data from all cell lines combined to identify miRNAs with a broad sensitizing effect. The miRNA-specific treatment effects, measured by tumor cell viability after treatment, were compared to the modal effect (across all miRNAs) and are indicated as relative effect size, as shown in Figure 1B. Due to our specific focus on the sensitization effect, our statistic model was established to detect a larger treatment effect, resulting in a one-side volcano plot. A false discovery rate (FDR) <0.1 indicates miRNAs with statistically significant treatment effects. Our discovery screen identified 55 miRNAs with significant sensitizing effect to thermoradiotherapy (Figure 1B; Table S1). While the modal effect shows a relative effect size of 0.00, miRNAs with significant sensitizing potential exhibit relative effect sizes ranging between −0.11 and −0.57 (Figure 1B). Consistent effects were observed across all four cervical cancer cell lines (Figure 1C). Notably, C-4 I and HeLa cells were more sensitive to thermoradiotherapy compared to CaSki and SiHa cells. The modal effect observed for each cell line aligns with the cell viability results of the negative control (NC), underscoring the robustness of the statistical model.

Validation screen reveals 18 miRNAs with significant sensitizing potential

The 55 candidate miRNAs identified in the discovery screen were subsequently validated in an independent set of 10 cancer cell lines, including 6 cervical cancer cell lines (778, 879, C-33A, DoTc2 4510, HT-3, and MS751) and 4 head and neck cancer cell lines (UM-SCC-47, VU-SCC-040, VU-SCC-120, and VU-SCC-147). Head and neck cancer was chosen because of the demonstrated efficacy of hyperthermia in a number of clinical trials.[1,25] Additionally, we aimed to explore whether the identified miRNAs exhibit sensitizing effects beyond cervical cancer.

Similar as in the discovery screen, we used 3D tumor spheroids for the validation screen and evaluated cell viability 3 days post-treatment (2 Gy irradiation and hyperthermia at 42°C for 60 min). In addition to the 55 candidate miRNAs, we selected 13 miRNAs from the discovery screen as "control" miRNAs, based on their relative effect size of approximately 0.00 (Table S1). These control miRNAs were used to calculate the effect size of the 55 candidate miRNAs. The analysis was conducted on the combined data of all cell lines and identified 18 miRNAs (miR-16, miR-22, miR-27a, miR-92a-1, miR-106b, miR-181a, miR-181c, miR-181d, miR-193a, miR-195, miR-221, miR-224, miR-330, miR-375, miR-455, miR-497, miR-1293, and miR-3158) with a significant sensitizing effect to thermoradiotherapy, demonstrating effect sizes ranging between −0.30 and −1.49 (Figure 2A). Importantly, the thermoradiosensitizing effect mediated by the 18 identified miRNAs demonstrated consistency across most cell lines, as illustrated for the top 5 identified miRNAs in Figure 2B.

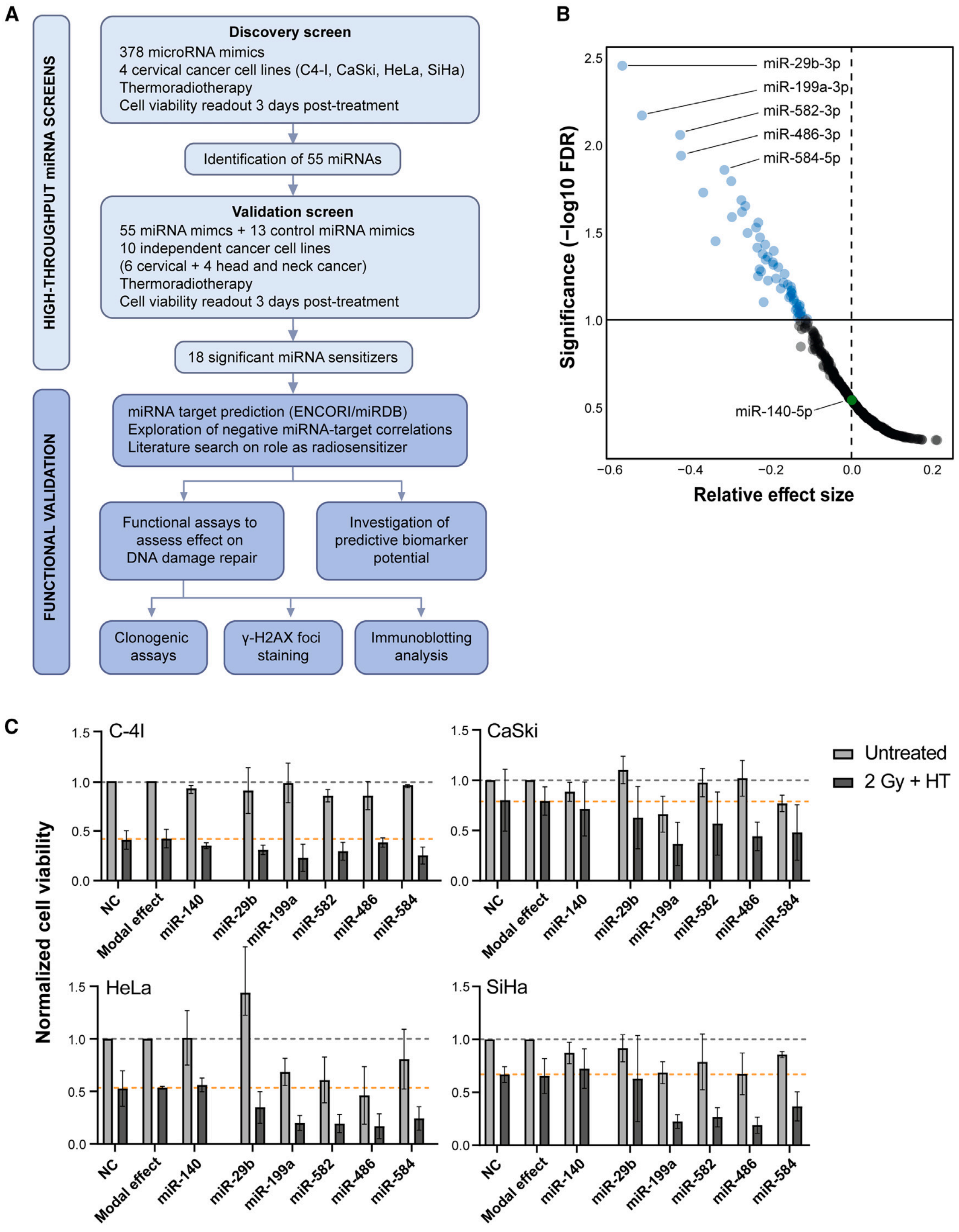

Figure 1. Overview of screen strategy and results of the discovery screen across four cervical cancer cell lines

(A) Schematic representation of the study outline. (B) Relative effect sizes obtained for each miRNA by comparison to the modal effect (across all miRNAs). miRNAs with a significant treatment effect (FDR <0.1; n = 55) are highlighted in blue. Additionally, miR-140-5p, with a relative effect size of approximately 0.00 is indicated in green. (C) Cell viability results normalized to negative control (NC; gray dotted line) for each cell line (C-4I, CaSki, HeLa, and SiHa), with the modal effect (across all miRNAs; orange dotted line), miR-140-5p as representative “control miRNA,” and the top 5 identified miRNAs indicated. Means ± SDs are shown. Untreated refers to miRNA transfected cells only, while 2 Gy+HT refers to cells that received thermoradiotherapy combined with miRNA transfection.

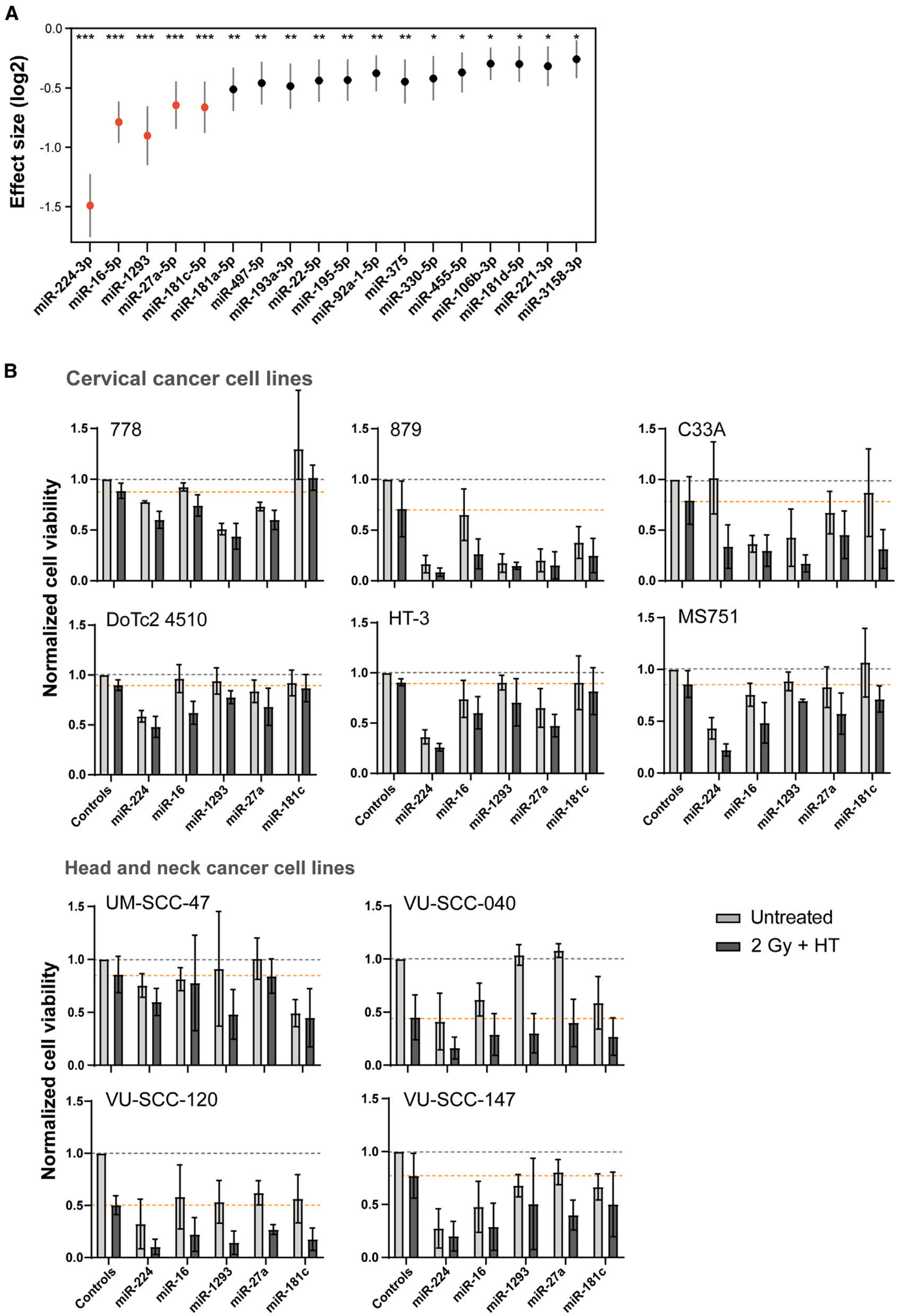

(legend on next page)

However, we observed variations in cell line sensitivity to thermoradiotherapy, with cell lines 879, VU-SCC-040 and VU-SCC-120 showing the strongest sensitivity. Notably, miR-224 demonstrated the strongest sensitizing effect in the majority of cell lines.

Identified miRNAs are associated with DNA repair pathways

Previous studies have shown that miRNAs can enhance sensitivity to ionizing radiation by targeting genes involved in DNA damage response.[26] To explore potential gene targets for the 18 miRNAs identified from our screens, we used the ENCORI or miRDB platform for miRNA target prediction,[27] followed by pathway analysis using the Database for Annotation, Visualization, and Integrated Discovery (DAVID) (Figure 3A).[28] Interestingly, pathway enrichment analysis using the predicted targets showed that the ataxia telangiectasia mutated (ATM)-dependent DNA damage response was one of the most significantly enriched pathways for 6 of the 18 miRNAs (Figure 3B; miR-16, miR-181c, miR-193a, miR-224, miR-375, and miR-497), supporting their involvement in inhibiting irradiation-induced double-strain break. For three other miRNAs (miR-27a, miR-330, and miR-455), we also found enrichment of the DNA damage response; however, this was not significant (Figures 3B and S1). Notably, miR-221 showed broad targeting ability on the DNA damage response, as demonstrated by the enrichment of pathways associated with the DNA damage response related to both ATM and ataxia telangiectasia and Rad3-related (ATR) kinases (Figure 3C). An overview of significantly enriched pathways for each miRNA is provided in Figure S1. Most of the predicted miRNA targets were associated with the two main DNA repair pathways: HR and non-homologous end joining (NHEJ) (Table 1). All other predicted targets linked to the DNA damage response are listed in Table S2.

Given that miRNAs operate by downregulating their gene targets, we computed correlations between the 18 identified miRNAs and genes associated with DNA repair pathways HR and NHEJ using publicly available genome-wide miRNA and messenger RNA (mRNA) expression profiles from cervical and head and neck cancers.[24,40] While each miRNA displayed at least one negative correlation, miR-22, miR-27a, miR-193a, miR-221, and miR-455 exhibited negative correlations with the majority of their predicted targets (Figure 3D).

Furthermore, a literature search was conducted that revealed supporting evidence for 7 of the 18 identified miRNAs in their role as radiosensitizers in cancer (Table 1). The 6 miRNAs (miR-16,[29–31] miR-22,[32] miR-181a,[33–35] miR-195,[36–39] miR-375,[41–44] and miR-497)[45–48] have been linked to radiosensitivity of cervical cancer and head and neck cancer, but also other cancer types such as breast cancer, colorectal cancer, esophageal and gastric cancer, glioblastoma, hepatocellular cancer, neuroblastoma, lung cancer, prostate cancer, and thyroid cancer (Table 1).

Thermoradiotherapy in combination with miRNAs results in reduced DNA damage repair

From the 18 identified miRNAs from the validation screen, 6 miRNAs were prioritized for subsequent functional validation. We selected miRNAs with the strongest sensitizing effect observed during the validation screen (effect size of <-0.5; miR-16, miR-27a, miR-181c, miR-224, and miR-1293). Additionally, miR-221 (effect size of −0.38) was included due to its numerous predicted targets associated with DNA repair pathways HR and NHEJ (Table 1).

We conducted clonogenic assays in SiHa cells to study cell survival following different treatment combinations, including miRNA mimic alone (untreated), irradiation (2 Gy) combined with miRNA mimics, and thermoradiotherapy (2 Gy and 42°C for 60 min) combined with miRNA mimics. We observed a significantly lower survival fraction when thermoradiotherapy was combined with miR-221, miR-224, or miR-1293 mimics compared to thermoradiotherapy combined with a NC (Figure 4A; representative images of the colony-forming assay are included in Figure S2). Upregulated expression of miRNA after transfection was measured using quantitative reverse-transcription PCR (RT-qPCR) (Figure S3B), with a fold change relative to the NC ranging from 10^2 to 10^8.

Furthermore, we investigated residual (unrepaired) DNA double-stranded breaks (DSBs) 24 h post-treatment using γ-H2AX foci staining. Thermoradiotherapy combined with either miR-16, miR-27a, miR-181c, miR-221, miR-224, or miR-1293 demonstrated a significant increase in DNA DSBs compared to the control, with the most pronounced increase observed for miR-221 (Figures 4B and 4C).

We subsequently performed western blot analysis to study the impact of miRNA modulation on the protein expression of several predicted targets associated with DNA repair pathways, specifically HR and NHEJ (Figures 4D and S4). Transfection of SiHa cells with miRNA mimics miR-16, miR-27a, miR-181c, miR-221, miR-224, or miR-1293 led to a reduction in ATM, a key player in HR. Regarding the regulation of NHEJ, we found strong effects for all six miRNAs on the protein expression of DNA-dependent protein kinase catalytic subunit (DNA-PKcs). In addition, we analyzed the protein expression of two other key players in NHEJ: Ku70 (gene name XRCC6) and Ku80 (gene name XRCC5). We observed that miR-1293 strongly reduced the protein expression of Ku80, while Ku70 expression was affected by miR-27a, miR-221, and miR-224. Additionally, these three miRNAs also reduced the expression of RAD51, a pivotal player in HR.

Figure 2. Results of the validation screen across 10 independent cervical and head and neck cancer cell lines
(A) Log2 effect sizes obtained for each miRNA by comparison to the control miRNAs (n = 13), with only significant miRNAs shown (18 out of 55; *$p < 0.05$; **$p < 0.001$; ***$p < 0.00001$). The top 5 miRNAs with the strongest sensitizing effect are highlighted in red. (B) Cell viability results for the top 5 identified miRNAs normalized to NCs (controls) on each cell line (untreated as gray dotted lines; 2 Gy+HT as orange dotted lines). Means ± SDs are shown. Statistical analysis of individual cell lines did not reveal significant differences, likely due to the limited number of samples per group (3 vs. 3). Untreated refers to miRNA transfected cells only, while 2 Gy+HT refers to cells that received thermoradiotherapy 1 day after miRNA transfection.

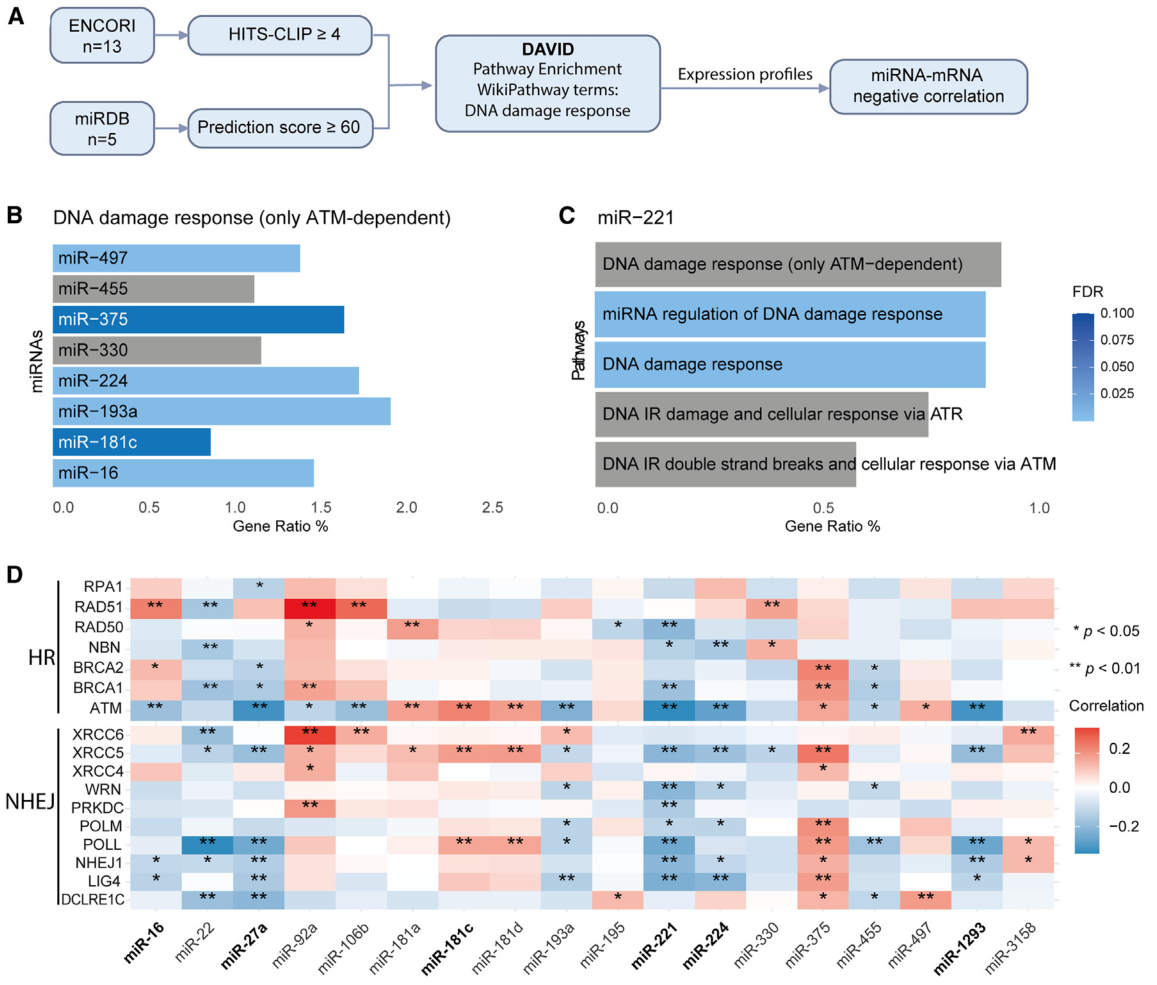

Figure 3. Overview of miRNA target prediction strategy, pathway enrichment, and correlation heatmap

(A) Flowchart of miRNA target prediction using the ENCORI (13 miRNAs) or miRDB (5 miRNAs) platform. (B) miRNAs that show significant enrichment for the DNA damage response. Gene ratio indicates the percentage of significant genes over the total genes associated with the DNA damage response. (C) The top 5 enriched pathways for miR-221 predicted targets. (D) Correlation heatmap illustrating miRNA-target co-expression in TCGA cervical cancer dataset for genes associated with DNA repair pathways HR and NHEJ. miRNAs selected for functional validation are indicated in bold. *$p < 0.05$; **$p < 0.01$. HITS-CLIP, high-throughput sequencing of RNA isolated by crosslinking immunoprecipitation.

miR-27a, miR-221, and miR-224 exhibit predictive biomarker potential for treatment response

Next, we explored the predictive biomarker potential of the identified miRNAs. To accomplish this, we utilized genome-wide miRNA expression profiles from advanced stage cervical cancer patients obtained from TCGA.[24] Unfortunately, TCGA dataset lacks information on cervical cancer patients who have undergone thermoradiotherapy, requiring us to focus on chemoradiotherapy instead. Using the clinical data from TCGA, we defined two groups of cancer patients based on their response to treatment: treatment-sensitive (n = 37), including those with complete and partial responses, and treatment-resistant (n = 18), including those with stable and progressive disease. Interestingly, we found that miR-27a, miR-221, and miR-224 are significantly downregulated in treatment-resistant cervical cancer patients (Figure 5), indicating their potential as predictive biomarkers for treatment response in cervical cancer patients.

DISCUSSION

Chemotherapy-based radiosensitization is the standard of care for treating locally advanced cervical and head and neck cancers. However, many patients cannot tolerate chemotherapy, and clinical trials have shown that thermoradiotherapy may be an equally effective alternative. miRNAs have previously been studied as potential targets for radiosensitization, but none of these studies have studied their potential role in thermoradiotherapy sensitization. In the present study, we identified and validated 18 miRNAs with significant sensitizing potential for thermoradiotherapy in 14 cervical and head and neck

Table 1. Overview of predicted miRNA targets associated with DNA damage repair pathways HR and NHEJ

	miR-16-5p	miR-22-5p	miR-27a-5p	miR-92a-1-5p	miR-106b-3p	miR-181a-5p	miR-181c-5p	miR-181d-5p	miR-193a-3p	miR-195-5p	miR-221-3p	miR-224-3p	miR-330-5p	miR-375	miR-455-5p	miR-497-5p	miR-1293	miR-3158-3p
HR																		
ATM			X				X			X	X							X
BRCA1							X				X							
MRE11							X				X							
NBN	X						X			X	X					X		
PAXIP1											X							
POLD3	X									X						X		
RAD50	X									X	X					X		
RAD51										X	X							
RAD52										X								
RAD54B		X																
NHEJ																		
LIG4							X											
NHEJ1										X								
POLL										X								
POLM							X			X								
PRKDC									X					X	X			
WRN		X									X							
XRCC5	X									X			X			X		
XRCC6											X							
Literature on radiosensitizer																		
	yes<br>CxCa[29]<br>NSCLC[30]<br>PCa[31]	yes<br>HCC[32]	no	no	no	yes<br>CRC[33]<br>CxCa[34]<br>GBM[35]	no	no	no	yes<br>BC[36]<br>CRC[37]<br>LC[38]<br>NSCLC[39]	no	no	no	yes<br>CxCa[41]<br>NB[42]<br>NSCLC[43]<br>OSCC[44]	no	yes<br>BC[45]<br>CxCa[46]<br>ESCC[47]<br>NSCLC[48]	no	no

Predicted targets of each miRNA (n = 18) are marked with an X, along with relevant literature supporting their role as radiosensitizers in cancer. BC, breast cancer; CRC, colorectal cancer; CxCa, cervical cancer; ESCC, esophageal squamous cell carcinoma; GBM, glioblastoma; HCC, hepatocellular cancer; HR, homologous recombination; LC, laryngeal cancer; NB, neuroblastoma; NHEJ, non-homologous end joining; NSCLC, non-small cell lung cancer; OSCC, oral squamous cell carcinoma; PCa, prostate cancer.

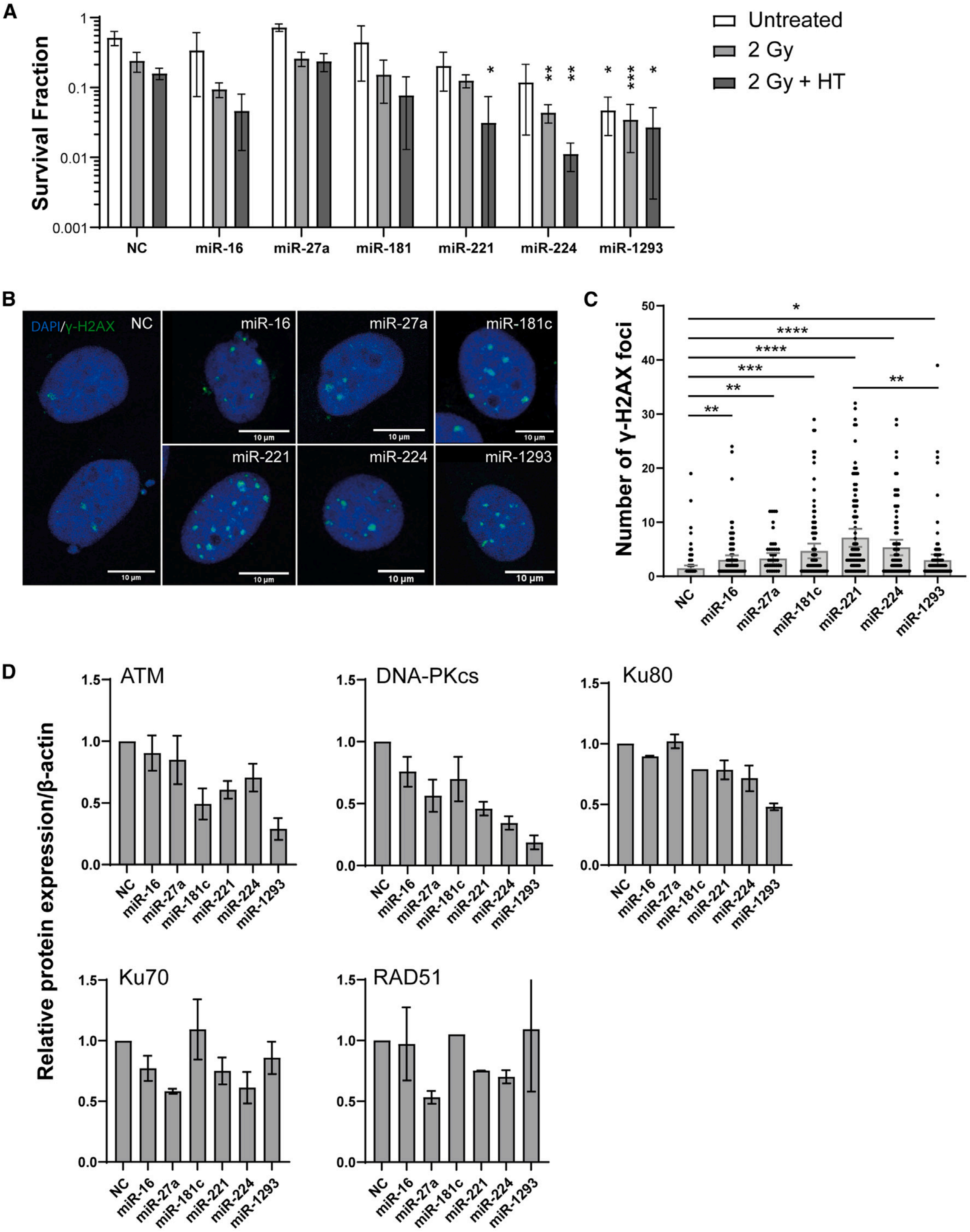

Figure 4. Response to irradiation (2 Gy), thermoradiotherapy (2 Gy+HT), and miRNA mimics in SiHa cells

(A) Clonogenic survival fractions for each miRNA are shown and compared to NC. (B) Representative images of γ-H2AX foci staining (in green) for each condition. (C) The number of γ-H2AX foci (the number of double-stranded breaks) 24 h after thermoradiotherapy combined with miRNA mimics or NC. Each graph represents the γ-H2AX foci in each cell, with a minimum of 70 cells per experiment. (D) Normalized protein expression against NC. Means ± SDs are shown. *$p < 0.05$; **$p < 0.01$; ***$p < 0.001$; ****$p < 0.0001$

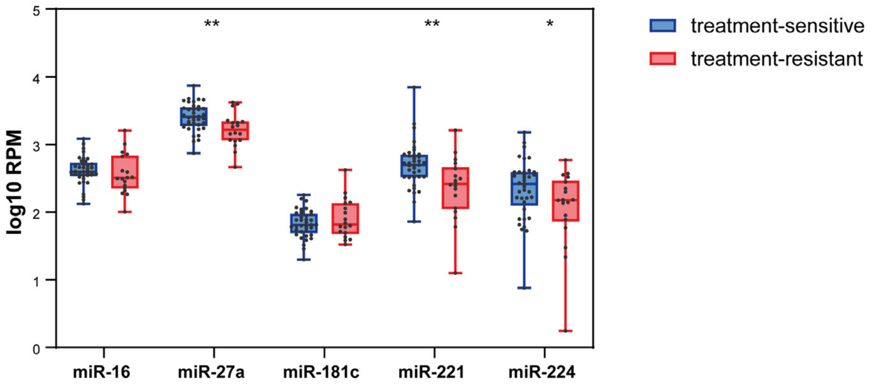

Figure 5. Expression profiles of miR-16, miR-27a, miR-181c, miR-221, and miR-224 in TCGA cervical cancer dataset

Cancer patients were divided into two groups based on their response to chemoradiotherapy: treatment sensitive (n = 37), including those with complete and partial responses, and treatment resistant (n = 18), including those with stable and progressive disease. *p < 0.05; **p < 0.01. RPM, reads per million.

cancer cell lines. Functional validation showed that miRNA-mediated sensitization was achieved by targeting genes associated with DNA repair pathways, including RAD51 (HR) and Ku80 (NHEJ).

An important aspect of this study is that we used a high-throughput miRNA screen on 3D spheroid models with cell viability as the readout, rather than conventional clonogenic assays to assess cell survival after treatment. Our approach overcomes the practical limitations of the clonogenic assay, including the long experiment duration, low sample throughput and time-consuming colony counting. The validity of our approach is supported by several findings. First, using the cellular ATP content as a measure of cell viability, we found a similar variation in sensitivity among cervical cancer cell lines and head and neck cell lines as reported by others using clonogenic assays.[49] This underscores the reliability of exploring treatment sensitization by measuring cell viability in a 3D model, while overcoming the difficulty when certain cell lines do not form colonies. Second, we observed similar sensitizing effects for most miRNAs when comparing results from clonogenic survival assays with cell viability results (Figure 4B). This is consistent with previous studies showing similar results between these two types of assays.[50,51] Third, among the discovered thermoradiosensitizing miRNAs, we identified several miRNAs previously described as potent radiosensitizers in cancer, including miR-16-5p, miR-181a-5p, miR-195-5p, miR-221-3p, miR-375-3p, and miR-497-5p (Table 1). Notably, several of these miRNAs have been described as radiosensitizers in cervical and/or head and neck cancers. For example, miR-16-5p has been shown to modulate the radiosensitivity of cervical cancer cells by regulating coactivator-associated arginine methyltransferase 1 (CARM1).[29] CARM1 plays a critical role in the DNA damage response by methylating p300, which enhances the interaction between p300 and BRCA1 (key player in HR), thereby promoting BRCA1 transcriptional activity.[52] Furthermore, miR-181a-5p was found to target Raf kinase inhibitor protein in nasopharyngeal cancer cells thereby enhancing radiotherapy resistance.[53] In addition, Ke and colleagues found that miR-181a-5p can confer radiotherapy resistance in cervical cancer cells by targeting pro-apoptotic protein kinase C delta (PRKCD) and that its expression may serve as a prognostic biomarker for radiotherapy response.[34]

On top we observed mostly consistent sensitizing effects in different cancer types (cervical and head and neck cancer), highlighting the potential broad applicability of these miRNAs in improving therapeutic outcomes. Some observed differences between cell lines are most likely due to inherent differences in radiation sensitivity and/or differences in endogenous miRNA levels between cell lines (Figure S3A).[54]

Interestingly, Western blot analysis revealed downregulation of ATM and DNA-PKcs (gene name *PRKDC*) after transfection of all six miRNAs (Figure 4D). As early sensors of DNA DSBs, ATM and DNA-PKcs activate a series of kinase reactions essential for DNA DSB repair.[55,56] Therefore, the downregulation of ATM and DNA-PKcs could inhibit DNA damage repair, which is consistent with our findings from γ-H2AX stainings. However, the types of interactions may vary among the miRNAs. The negative correlation in expression levels between three miRNAs (miR-27a, miR-181c, and miR-221) and ATM was confirmed by TCGA data (Figure 3D). Target prediction using a combination of computational and experimental strategies (high-throughput sequencing of RNA isolated by crosslinking immunoprecipitation [HITS-CLIP] data) also supports the interaction between these miRNAs and their targets, suggesting that ATM is directly targeted by miR-27a, miR-181c, and miR-221. For DNA-PKcs, although TCGA data show a negative correlation between the miRNAs and PRKDC, target prediction did not provide evidence for a direct interaction between the miRNAs and PRKDC, suggesting an indirect effect of the miRNAs.

Repair of radiation-induced DNA DSBs is typically accomplished by two pathways: NHEJ and HR.[57] During classical NHEJ, DNA DSBs are initially recognized by the Ku70-Ku80 heterodimers, which recruit and promote the binding of other NHEJ proteins, including DNA-PKcs, ligase 4, and XRCC4.[58] Thus, downregulation of Ku70 (observed with miR-16, miR-181c, miR-221, miR-224, and miR-1293) and Ku80 (observed with miR-16, miR-27a, miR-221, miR-224, and miR-1293) could inhibit NHEJ. While miR-16[29] and miR-1293[59] have been reported to target DNA repair genes, their effects on Ku70 and Ku80 have not been previously demonstrated. Target prediction supports miR-16 targeting of Ku80 (XRCC5), but the mechanism by which miR-1293 inhibits DNA damage repair requires further investigation.

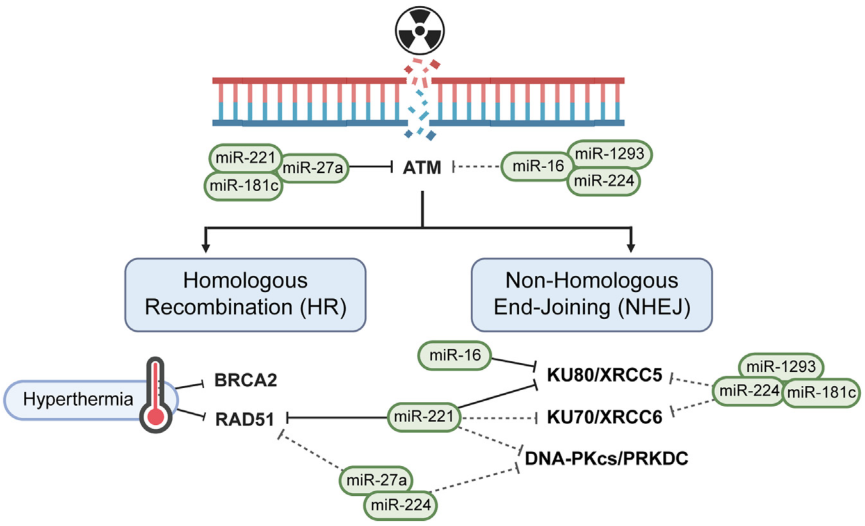

Figure 6. Network model illustrating our proposed mechanism of miRNA-mediated sensitization to thermoradiotherapy based on the results of this study

Direct regulation is indicated by black solid line, and indirect regulation is indicated by gray dotted line.

Alternatively, DNA DSBs can also be repaired by HR. Following initiation resection by the MRN complex (MRE11-RAD51-NBS1) and activation of ATM, HR is catalyzed by several enzymes, in particular by recombinase RAD51.[60] RAD51 plays an important role in HR by assembling filament on DNA, interacting with other proteins, and then facilitating HR.[61] In our validation, miR-221 mimic transfection resulted in a pronounced downregulation of RAD51 protein levels. This observation is further supported by computational and experimental target prediction, suggesting that miR-221 may inhibit HR by directly affecting RAD51 levels (Figure 3D). These results are summarized in a network model shown in Figure 6, which illustrates our proposed mechanism of miRNA-mediated sensitization of thermoradiotherapy.

One limitation of our study is the use of single-dose irradiation (2 Gy) instead of applying a clinically relevant treatment regimen. For locally advanced cervical cancer, the clinical treatment regimen typically involves fractionated radiotherapy with 20–25 fractions and a total dose of 45–50 Gy.[10] However, single-dose irradiation is often used in *in vitro* studies to simplify experimental conditions and allow for controlled analysis of cellular responses to radiation.[49] Future studies using fractionated radiation may provide more clinically relevant insights and potentially confirm our findings. Additionally, our functional validation was conducted exclusively in cervical cancer cell lines. Expanding validation to other cancer cell lines, particularly head and neck cancer cells, as well as non-malignant cells, may strengthen our findings and provide valuable insights into the toxicity profile of the miRNAs. miRNA toxicity is a critical issue that could significantly impact therapeutic efficiency, making it essential to evaluate the toxicity profile of each miRNA. In this study, we used a 2-nM miRNA mimic concentration for each cell line, which was selected based on prior optimization efforts.[23,62] While miR-224 exhibited the strongest sensitizing effects across cell lines, it also notably reduced cell viability before treatment (Figure 2B), suggesting general toxicity. To address this, we tested a lower concentration of miR-224 and found that it reduced its impact on cell viability (Figure S5), particularly in HeLa and SiHa cells, demonstrating a dose-dependent effect. A similar trend was observed for miR-16, miR-27a, and miR-1293. These findings highlight the need for further optimization of miRNA mimic concentrations in future studies to minimize potential toxicity while maintaining therapeutic efficacy.

While this study primarily focused on miRNA-mediated modulation of the two main DNA repair pathways, HR and NHEJ, other DNA damage response-associated genes remain largely unexplored in this context. Additionally, our pathway analysis revealed several other significantly enriched pathways, including those related to angiogenesis, apoptosis, cell-cycle regulation and immune responses, as shown in Figure S1. Further investigation into these pathways and other DNA damage response-associated genes may provide additional leads for therapeutic intervention and could further advance our understanding of miRNA-mediated treatment sensitization.

The field of miRNA-based therapeutics has gained significant interest due to the central role of miRNAs in gene regulation and their involvement in various cancers. Several miRNA mimics and inhibitors, such as miR-10b, miR-16, miR-34a, miR-155, and miR-193a, have been or are currently being investigated in clinical trials for the treatment of cancer and other diseases.[63,64] Unlike single-gene therapies, miRNA-based therapeutics offer a unique advantage due to their ability to simultaneously target multiple pathways (i.e., HR and NHEJ). However, despite promising preclinical results, the translation of miRNA therapeutics into clinical practice remains highly challenging.[65]

One major hurdle of miRNA therapeutics is the difficulty in achieving efficient and specific delivery. miRNA mimics and inhibitors require stable delivery systems to prevent degradation and ensure targeted cellular uptake, yet existing strategies often lead to off-target effects, immune activation, or insufficient bioavailability. Early clinical trials, such as a phase 1 trial for MRX34 (a miR-34a mimic; this study was registered at ClinicalTrials.gov: NCT01829971),[66] a phase 2 trial for lademirsen (a miR-21 inhibitor; this study was registered at ClinicalTrials.gov: NCT02855268),[67] and phase 2 trials for Miravirsen (a miR-122 inhibitor; these studies were registered at ClinicalTrials.gov: NCT01872936 and NCT02031133)[68] encountered setbacks due to severe immune-mediated toxicities or limited efficacy, ultimately leading to trial discontinuation. These examples highlight the complexities of miRNA-based therapeutics, particularly regarding safety and specificity.

Nanoparticle-based delivery systems have shown promise in overcoming some of these challenges by enhancing stability and targeting efficiency.[64] In the context of cervical cancer, localized delivery approaches could mitigate systemic toxicity.[69] However, while the anatomical accessibility of the cervix may facilitate localized administration, this does not inherently resolve broader delivery challenges, including endosomal sequestration and biodistribution concerns. Furthermore, tumor heterogeneity, hypoxic environments, and variable miRNA expression profiles within cervical cancer further complicate therapeutic efficacy and patient stratification.

Despite these challenges, miRNA therapeutics continue to hold potential, particularly when integrated into multimodal treatment strategies. For example, in thermoradiotherapy, hyperthermia enhances tumor vasculature permeability, which could improve nanoparticle uptake and facilitate miRNA-based radiosensitization.[70] However, preclinical and clinical studies are needed to determine the safety and efficacy of such approaches.

In addition to their role as therapeutic agents, this study has identified several miRNAs that show potential as predictors of treatment response in cervical cancer patients. Specifically, miR-27a, miR-221, and miR-224 show significant downregulation in treatment-resistant patients, suggesting their utility as predictive markers of treatment response. In support of these findings, Ren and colleagues demonstrated that miR-27a plays a critical role in modulating the radiosensitivity of triple-negative breast cancer cells, suggesting that miR-27a expression could serve as a valuable marker to identify patients who will benefit from radiotherapy treatment.[71] In addition, Upraity et al. found that increased miR-224 expression increased the radiosensitivity of glioblastoma cells.[72] Notably, TCGA cervical cancer dataset used in our study only includes patients treated with radiotherapy or chemoradiotherapy. While our study focuses on thermoradiotherapy, the potential biomarker utility of the identified miRNAs in the context of chemoradiotherapy underscores their broad applicability. This suggests that our findings could be extrapolated to other combined-modality treatments that include radiotherapy. Further validation studies in larger patient cohorts and in other cancer types are warranted to confirm the predictive value of miR-27a, miR-221, and miR-224 and to explore their applicability in clinical practice.

In conclusion, this study highlights the potential role of miRNAs as sensitizers of thermoradiotherapy and improves our understanding of the molecular mechanisms underlying miRNA-mediated sensitization of thermoradiotherapy. Further research on the clinical translation of these findings, including prospective validation studies and mechanistic investigations, is warranted to fully exploit the therapeutic potential of miRNA-based approaches in combination with thermoradiotherapy for the treatment of locally advanced solid tumors.

MATERIALS AND METHODS

Cell lines and cell culture

Human cervical cancer cell lines C-33A, C-4 I, CaSki, DoTc2 4510, HeLa, MS751, and SiHa were obtained from the American Type Culture Collection (ATCC, Manassas, VA) and cultured in Dulbecco's modified Eagle's medium (DMEM) formulated with high glucose and L-glutamine (Thermo Fisher Scientific, Waltham, MA) and supplemented with 10% fetal bovine serum (FBS), 100 U/mL penicillin, and 100 μg/mL streptomycin (all from Thermo Fisher Scientific). Human cervical cancer cell line HT-3 was obtained from the ATCC and cultured in McCoy's 5a medium supplemented with 10% FBS, 100 U/mL penicillin, and 100 μg/mL streptomycin (all from Thermo Fisher Scientific). Two cervical cancer cell lines, 778 and 879, were previously provided by Dr. P.L. Stern (Paterson Institute for Cancer Research, Manchester, UK) and cultured in RPMI 1640 medium (Thermo Fisher Scientific) supplemented with 10% FBS, penicillin at 100 U/mL, and streptomycin at 100 μg/mL (all from Thermo Fisher Scientific).[73] Head and neck squamous cell carcinoma (HNSCC) cell lines UM-SCC-47, VU-SCC-040, VU-SCC-120, and VU-SCC-147 were obtained as described previously and cultured with DMEM supplemented with 10% FBS, 1 mM sodium pyruvate, 100 U/mL penicillin, and 100 μg/mL streptomycin (all from Thermo Fisher Scientific).[74–76] Cell cultures were maintained at 37°C in a humidified atmosphere with 5% CO_2. Cell lines were authenticated by short-tandem repeat (STR) testing, monthly tested for the presence of mycoplasma and HPV status was confirmed using the QIAscreen HPV PCR assay (Qiagen, Hilden, Germany).

Irradiation and hyperthermia

Cells were irradiated with 2 Gy of γ-radiation at room temperature using the Gammacell 220 Cobalt irradiator with a ^{131}Cs source (MDS Nordion, Ottawa, Canada). Hyperthermia treatment of cells started within 30 min after irradiation. Incubation of cells was performed by partially submerging the plates in a thermostatically controlled water bath (VWR International, Amsterdam, the Netherlands) for 60 min at 42°C (±0.1°C). The temperature was measured in parallel plates with a thermometer, and the desired temperature was reached in approximately 8 min for 384-well plates and 5 min for 6-well plates.

High-throughput miRNA screens

For the discovery screen, four cervical cancer cell lines (C-4 I, CaSki, HeLa, and SiHa) were subjected to high-throughput reverse transfection using 384-well ultra-low attachment plates (Corning, New York, NY) to allow for 3D cell cultures (spheroids). A total of 378 miRNAs (Table S1) were selected based on in-house and publicly available genome-wide miRNA expression data from cancer types relevant to our study (i.e., cervical cancer, head and neck cancers) as well as literature supporting their role as a radiosensitizer in cancer.[22,24,40,77,78] Cells were transfected using an optimized reverse transfection protocol, in which efficiency was carefully assessed through miRNA qPCR, miRNA target western blot analysis, and cellular viability measurements, as described previously.[23,62] In brief, on day 0 transfection complexes were prepared by mixing 0.04 μL DharmaFECT4 transfection reagent (Horizon Discoveries, Cambridge, UK) and 2 nM (end concentration) of miRNA mimic (miRIDIAN miRNA mimic library version 19 [Horizon Discoveries]) with 20 μL Opti-MEM serum-free medium (Thermo Fisher Scientific). Cells were harvested, resuspended in culture medium, and plated in a 384-well format (500

cells/20 μL/well) simultaneously with the addition of transfection complexes. Plates were sealed with breathable film to prevent evaporation. On day 1, cells were treated with thermoradiotherapy or kept in culture as a control. Cell viability was assessed on day 4 by adding 15 μL Cell-Titer Glo 3D (Promega, Madison, WI) per well, after which luminescence was measured using an Infinite F200 microplate reader (Tecan Group, Männedorf, Switzerland). The discovery screen was performed in triplicate.

For the validation screen, 10 additional cancer cell lines were utilized, including 6 cervical cancer cell lines (778, 879, C-33A, DoTc2 4510, HT-3, and MS751) and 4 HNSCC cell lines (UM-SCC-47, VU-SCC-040, VU-SCC-120, and VU-SCC-147). From the discovery screen, 55 candidate miRNAs along with 13 control miRNAs were selected for validation. The setup and readout of the validation screen was similar to that of the discovery screen, including the amount of cells, transfection reagent, and miRNA mimic concentration. The validation screen was performed in triplicate.

miRNA transfection for functional assays

SiHa cells were reverse transfected with miRNA mimics for hsa-miR-16-5p, hsa-miR-27a-5p, hsa-miR-181c-5p, hsa-miR-221-3p, hsa-miR-224-3p, hsa-miR-330-5p, hsa-miR-1293, or NC #2 (C-300483-03, C-301028-01, C-300556-03, C-300578-05, C-301304-00, C-301082-01, C-301347-00, CN-002000-01 [Horizon Discoveries]) in ultra-low attachment plates following the same protocol outlined for the miRNA screens. In brief, transfection complexes were prepared by mixing 2 μL (end volume 0.04 μL) DharmaFECT4 transfection reagent (Horizon Discoveries) and 2 nM (end concentration) of miRNA or control mimic with 200 μL Opti-MEM serum-free medium (Thermo Fisher Scientific). Harvested cells were plated in 6-well ultra-low attachment plates (Corning) at a density of 85,000 cells/1.8 mL/well.

RNA isolation

Total RNA was isolated from cells using the miRNeasy Tissue/Cells Advanced kit, including on-column DNase treatment, according to the manufacturer's instructions (Qiagen). RNA concentrations were measured using NanoDrop One (Thermo Fisher Scientific).

RT-qPCR

This study was performed in compliance with the minimum information for publication of qPCR experiments guidelines, where applicable.[79] Reverse transcription was performed using the TaqMan microRNA Reverse Transcription kit (4366596; Thermo Fisher Scientific). The manufacturer's protocol was adapted to multiplex reverse transcription for all miRNAs and the reference gene, as described previously.[77,80] In brief, 50 ng total RNA was reverse transcribed in 16.5-μL reactions containing 9 μL primer pool, 0.45 μL deoxynucleotide triphosphate (100 mM), 2.25 μL 10× RT buffer, 0.3 μL RNase inhibitor (20 U/μL), and 4.5 μL MultiScribe Reverse Transcriptase. Expression of hsa-miR-16-5p, hsa-miR-27a-5p, hsa-miR-181c-5p, hsa-miR-221-3p, hsa-miR-224-3p, hsa-miR-330-5p, and hsa-miR-1293 was determined using TaqMan microRNA assays (000391, 002445, 000482, 000542, 121210_mat, 002230, 002905; Thermo Fisher Scientific). U75 was included as a reference gene (001219; Thermo Fisher Scientific). qPCR was performed in 10-μL reactions containing 5 μL TaqMan Universal Master Mix II, no UNG (4440043; Thermo Fisher Scientific), 0.5 μL miRNA-specific TaqMan microRNA assays (catalog numbers for each miRNA assay are listed above), 3.5 μL H_2O, and 1 μL cDNA. qPCR reactions were performed on the ABI 7500 Fast Real-Time PCR System (4351107; Thermo Fisher Scientific) using the following conditions; 5 min at 95°C followed by 40 cycles of 15 s at 95°C and 1 min at 60°C. H_2O was tested as a negative PCR control. qPCR analysis and determination of Ct values were conducted using Applied Biosystem 7500 Software version 2.3 (4397808; Thermo Fisher Scientific). The miRNA expression levels were normalized to U75 by applying the $2^{-\Delta Ct}$ method.[81]

Clonogenic assay

Clonogenic assays were performed as described previously.[82,83] Cells were collected 2 days post-miRNA transfection (to harvest enough cells for the subsequent protocol) and plated at different densities depending on the treatment group (50, 100, 200, 500, 1,000 or 2,000 cells/6-well) 4–8 h prior to treatment. Subsequently, cells were treated with either irradiation (2 Gy) alone or thermoradiotherapy (2 Gy and 42°C for 60 min) and incubated for 10–12 days to allow for colony formation. The plates were stained with a methanol-crystal violet solution (5 mg/mL crystal violet in 10% ethanol, 50% methanol, and 40% water) followed by scanning and counting with GelCount (Oxford Optronix, Abington, UK). Plating efficiencies were defined by dividing the amount of counted colonies by the amount of seeded cells. Survival fractions were calculated by normalization to the plating efficiency of untreated cells.

γ-H2AX immunofluorescence assay

SiHa cells were reverse transfected in the 8-well Nunc Lab-Tek II Chamber Slide system (Thermo Fisher Scientific) by mixing 0.6 μL (end volume 0.04 μL) DharmaFECT4 transfection reagent (Horizon Discoveries) and 2 nM miRNA or control mimic with 80 μL opti-MEM serum-free medium (Thermo Fisher Scientific). Harvested cells were seeded at 9,000 cells/520 μL/well. Subsequently, cells were treated with ionizing radiation (2 Gy) and hyperthermia (42°C for 60 min) and 24 h post-treatment the cells were fixed with 2% paraformaldehyde and permeabilized with 0.1% Triton X-100 in phosphate-buffered saline (PBS). Slides were then incubated with 1:200 Alexa Fluor 488 anti-H2A.X Phospho (Ser139) antibody (catalog no. 613405; BioLegend, San Diego, CA) and counterstained with 1 μg/mL DAPI. Slides were then visualized with a Nikon A1R HD25 confocal microscope (Nikon Europe, Amstelveen, the Netherlands). H2AX foci numbers were counted manually and at least 70 cells per condition per experiment were scored in three independent experiments.

Automated western blot

SiHa cells were collected 24 h post-miRNA transfection and plated in 6-well plates at a density of 85,000 cells/well 4–8 h prior to treatment. Cells were treated with irradiation (2 Gy) and hyperthermia (42°C for 60 min), and 4 h post-treatment cells were harvested. Whole-cell lysates were prepared on ice using the RIPA Lysis Buffer System

(Santa Cruz Biotechnology, Dallas, TX), according to the manufacturer's instructions. Protein concentrations were determined using Pierce bicinchoninic acid protein assay following the manufacturer's instructions. The automated western blots were conducted using JESS Simple Western instrument (ProteinSimple, Bio-Techne, Minneapolis, MN). Proteins (3 μg) were diluted in 0.1× sample buffer and 1× fluorescent master mix (EZ standard pack; Bio-Techne) to a total volume of 6 μL/well and separated using a 12- to 230-kDa or a 66- to 440-kDa separation module (Bio-Techne). Primary antibodies were diluted in antibody diluent 2 (Bio-Techne) and included rabbit anti-ATM (1:50, catalog no. 2873; Cell Signaling Technologies, Danvers, MA), mouse anti-β-actin (1:12.5, catalog no. 3700s; Cell Signaling Technologies), rabbit anti-DNA-PKcs (1:100, bsm-52493R, Bioss, Woburn, MA), rabbit anti-Ku70 (1:500, catalog no. PA5-27538, Thermo Fisher Scientific), rabbit anti-Ku70/Ku80 (1:500, catalog no. PA-5-27538; Thermo Fisher Scientific), and mouse anti-RAD51 (1:50, catalog no. MS-988-P1; Thermo Fisher Scientific). Secondary antibody detection modules anti-mouse and anti-rabbit were used according to the manufacturer's instructions. The RePlex module (Bio-Techne) was used to allow sequential detection of multiple proteins. Data were analyzed using the software Compass for Simple Western version 6.3.

miRNA target prediction

Target prediction of selected miRNAs from the validation screen (n = 18) was performed using the ENCORI platform version 2.0[27] or miRDB (https://mirdb.org).[84] The ENCORI platform was employed for 13 miRNAs (miR-16-5p, miR-106b-5p, miR-181a-5p, miR-181c-5p, miR-181d-5p, miR-193a-5p, miR-195-5p, miR-221-3p, miR-224-3p, miR-330-5p, miR-375, miR-455-5p, and miR-497-5p). No predicted targets were identified for 5 miRNAs (miR-22-5p, miR-27a-5p, miR-92a-1-5p, miR-1293, and miR-3158-3p) using the ENCORI platform, leading us to utilize miRDB for target prediction. Targets predicted by ENCORI with a HITS-CLIP number $\geq$4 and targets by miRDB with a prediction score $\geq$60 were subjected to pathway analysis using DAVID version 2024q2,[28] with WikiPathways version 20241110 as reference. Pathways associated with the DNA damage response were selected for further investigation.

External expression data of cervical tissue specimens

Genome-wide miRNA and mRNA expression profiles along with clinical data from cervical cancer patients[24] and head and neck cancer patients[40] was obtained from TCGA. Pearson correlation coefficients (r) were computed between miRNAs and their predicted miRNA targets and visualized in a correlation heatmap. Patients with advanced cervical cancer were classified into two groups according to their response after chemoradiotherapy: treatment sensitive (n = 37), including complete and partial response, or treatment resistant (n = 18), including stable and progressive disease.

Statistical analysis

Analysis of high-throughput screens

As the number of cell lines was relatively small in the discovery screen, we opted for a simple analysis model per miRNA: a linear mixed effect model for the $\log_2$(response), with thermoradiotherapy treatment and cell lines as fixed factors and assay (biological replicates) as a random effect.[85] miRNA-specific treatment effects were contrasted with the modal effect (across all miRNAs) to filter out effects that are mostly due to the treatment alone. Hence, resulting miRNAs have, in conjunction with the treatment, a significantly larger effect than the modal effect. An FDR of $\leq$0.10 was used as threshold for significance. The discovery screen data was analyzed using the R-package ShrinkBayes version 2.13.7, which enables efficient estimation for large screens and small sample sizes.[85] From the results of the discovery screen, 13 miRNAs were designated as control miRNAs based on their relative effect size (around 0.0; Table S2).

For the validation screen, the utilization of a larger number of cell lines allowed for a slightly more complex model, also to disentangle the treatment and miRNA effect. For each miRNA, a linear mixed-effects model was used on the $\log_2$(response) using miRNA presence (yes/no), thermoradiotherapy treatment (yes/no), cancer type (cervical cancer/HNSCC), and HPV status (negative/positive) as fixed effects (the latter two as potential confounders), plus cell line, assay (biological replicates nested within cell line), and pair (nested within assay) as random effects. The first accounts for variation across cell lines (now modeled as random effects as we have 11 levels), the second for correlation between technical repeats of screens, and the last accounts for correlation between responses of the parental (no miRNA) and non-parental settings within one screen. This model was fit for both control miRNAs and the candidate miRNAs were corrected for the mean effect of the control miRNAs (by subtracting the latter from the responses of the candidate miRNAs) to ensure that selected miRNAs have significantly stronger effects than the control miRNAs. Significance of the miRNA was tested using a likelihood ratio test (chi-squared; df = 1) of the model against the null model, which contains all factors except for the miRNA presence covariate. Likewise, treatment-miRNA interactions were tested by adding such an interaction to the model and testing it against the model with main effects only. Resulting p values were Bonferroni corrected for multiple testing, and effect size estimates were back-transformed to the original scale, rendering fold changes. The R-package lme4 version 1.1–35 was used to fit the mixed models.[86]

Analysis of functional assays

All data are expressed as mean ± SD. The statistical significance of clonogenic assays, γ-H2AX immunofluorescence assays, and immunoblotting was tested using the Kruskal-Wallis test with the post hoc Dunn's test for comparison to NC within the same treatment group (untreated, 2 Gy or 2 Gy+HT). Comparison of miRNA expression profiles between radio-sensitive and radio-resistant groups was made using the Mann-Whitney test (*$p < 0.05$; **$p < 0.01$; ***$p < 0.001$; ****$p < 0.0001$). Correlation between miRNA and mRNA expression was tested using Spearman correlation (*$p < 0.05$; **$p < 0.01$). Analyses of functional experiments were performed with GraphPad Prism version 10 (GraphPad Software, La Jolla, CA). Statistical analyses of publicly available data were performed with R (version 4.2.1, https://www.R-project.org/). Data processing and

figures were generated using the readr (version 2.1.5), dplyr (version 1.1.4) and ggplot2 (version 3.4.4) packages. Study outline and network model were illustrated using BioRender (https://www.biorender.com/) and Adobe Illustrator 2023.

DATA AVAILABILITY

mRNA and miRNA expressions and treatment response information are available from TCGA dataset. All other data supporting the findings of this study are available from the corresponding author upon reasonable request.

ACKNOWLEDGMENTS

We would like to thank A. Jaspers and A.P. van Splunter for their excellent technical assistance. This research was supported by Cancer Center Amsterdam grant no. CCA2019-9-59 and the China Scholarship Council grant no. 202008420215.

AUTHOR CONTRIBUTIONS

Conceptualization: B.C.S., R.D.M.S., A.L.O., L.J.A.S., and V.W.v.B. Methodology: M.X., B.C.S., R.D.M.S., and A.H. Formal analysis & investigation: M.X., B.C.S., M.A.v.d.W., and D.M. Visualization: M.X. and B.C.S. Writing – original draft: M.X., B.C.S., and M.A.v.d.W. Writing – review & editing: B.C.S., M.X., R.D.M.S., A.H., A.L.O., L.J.A.S., and V.W.v.B. Funding acquisition: B.C.S. and M.X. Supervision: B.C.S. and R.D.M.S. All authors have read and agreed to the published version of the manuscript.

DECLARATION OF INTERESTS

R.D.M.S. has a minority stake in Self-screen, a spin-off company of VU University Medical Center Amsterdam. V.W.v.B. is a named inventor on issued patents licensed to ORCA Therapeutics and owns options on shares of ORCA Therapeutics.

SUPPLEMENTAL INFORMATION

Supplemental information can be found online at https://doi.org/10.1016/j.omtn.2025.102500.

REFERENCES

1. Datta, N.R., Ordóñez, S.G., Gaipl, U.S., Paulides, M.M., Crezee, H., Gellermann, J., Marder, D., Puric, E., and Bodis, S. (2015). Local hyperthermia combined with radiotherapy and-/or chemotherapy: recent advances and promises for the future. Cancer Treat Rev. *41*, 742–753.
2. Lutgens, L.C.H.W., Koper, P.C.M., Jobsen, J.J., van der Steen-Banasik, E.M., Creutzberg, C.L., van den Berg, H.A., Ottevanger, P.B., van Rhoon, G.C., van Doorn, H.C., Houben, R., and van der Zee, J. (2016). Radiation therapy combined with hyperthermia versus cisplatin for locally advanced cervical cancer: Results of the randomized RADCHOC trial. Radiother. Oncol. *120*, 378–382.
3. van der Zee, J., González González, D., van Rhoon, G.C., van Dijk, J.D., van Putten, W.L., and Hart, A.A. (2000). Comparison of radiotherapy alone with radiotherapy plus hyperthermia in locally advanced pelvic tumours: a prospective, randomised, multicentre trial. Dutch Deep Hyperthermia Group. Lancet *355*, 1119–1125.
4. Mitsumori, M., Zeng, Z.F., Oliynychenko, P., Park, J.H., Choi, I.B., Tatsuzaki, H., Tanaka, Y., and Hiraoka, M. (2007). Regional hyperthermia combined with radiotherapy for locally advanced non-small cell lung cancers: a multi-institutional prospective randomized trial of the International Atomic Energy Agency. Int. J. Clin. Oncol. *12*, 192–198.
5. Datta, N.R., and Bodis, S. (2019). Hyperthermia with radiotherapy reduces tumour alpha/beta: Insights from trials of thermoradiotherapy vs radiotherapy alone. Radiother. Oncol. *138*, 1–8.
6. Crezee, J., Franken, N.A.P., and Oei, A.L. (2021). Hyperthermia-Based Anti-Cancer Treatments. Cancers *13*, 1240.
7. Bodis, S., Ghadjar, P., and van Rhoon, G. (2022). Oncologic Thermoradiotherapy: Need for Evidence, Harmonisation, and Innovation. Cancers *14*, 2418.
8. Paulides, M.M., Verduijn, G.M., and Van Holthe, N. (2016). Status quo and directions in deep head and neck hyperthermia. Radiat. Oncol. *11*, 21.
9. Mei, X., Kok, H.P., Rodermond, H.M., van Bochove, G.G.W., Snoek, B.C., van Leeuwen, C.M., Franken, N.A.P., Ten Hagen, T.L.M., Crezee, J., Vermeulen, L., et al. (2023). Radiosensitization by Hyperthermia Critically Depends on the Time Interval. Int. J. Radiat. Oncol. Biol. Phys. *118*, 817–828.
10. IJff, M., Crezee, J., Oei, A.L., Stalpers, L.J.A., and Westerveld, H. (2022). The role of hyperthermia in the treatment of locally advanced cervical cancer: a comprehensive review. Int. J. Gynecol. Cancer *32*, 288–296.
11. Adam, J.A., Arkies, H., Hinnen, K., Stalpers, L.J., van Waesberghe, J.H., Stoker, J., van Os, R., Laan, J.J., Mom, C.H., and van Eck-Smit, B.L. (2018). 18F-FDG-PET/CT guided external beam radiotherapy volumes in inoperable uterine cervical cancer. Q. J. Nucl. Med. Mol. Imaging *62*, 420–428.
12. Hurwitz, M., and Stauffer, P. (2014). Hyperthermia, radiation and chemotherapy: the role of heat in multidisciplinary cancer care. Semin. Oncol. *41*, 714–729.
13. Elming, P.B., Sorensen, B.S., Oei, A.L., Franken, N.A.P., Crezee, J., Overgaard, J., and Horsman, M.R. (2019). Hyperthermia: The Optimal Treatment to Overcome Radiation Resistant Hypoxia. Cancers *11*, 60.
14. Dewhirst, M.W., Vujaskovic, Z., Jones, E., and Thrall, D. (2005). Re-setting the biologic rationale for thermal therapy. Int. J. Hyperther. *21*, 779–790.
15. Li, Z., Deng, J., Sun, J., and Ma, Y. (2020). Hyperthermia Targeting the Tumor Microenvironment Facilitates Immune Checkpoint Inhibitors. Front. Immunol. *11*, 595207.
16. Oei, A.L., Korangath, P., Mulka, K., Helenius, M., Coulter, J.B., Stewart, J., Velarde, E., Crezee, J., Simons, B., Stalpers, L.J.A., et al. (2019). Enhancing the abscopal effect of radiation and immune checkpoint inhibitor therapies with magnetic nanoparticle hyperthermia in a model of metastatic breast cancer. Int. J. Hyperther. *36*, 47–63.
17. Oei, A.L., van Leeuwen, C.M., ten Cate, R., Rodermond, H.M., Buist, M.R., Stalpers, L.J.A., Crezee, J., Kok, H.P., Medema, J.P., and Franken, N.A.P. (2015). Hyperthermia Selectively Targets Human Papillomavirus in Cervical Tumors via p53-Dependent Apoptosis. Cancer Res. *75*, 5120–5129.
18. Wang, L., Zhan, G., Maimaitiyiming, Y., Su, Y., Lin, S., Liu, J., Su, K., Lin, J., Shen, S., He, W., et al. (2022). m(6)A modification confers thermal vulnerability to HPV E7 oncotranscripts via reverse regulation of its reader protein IGF2BP1 upon heat stress. Cell Rep. *41*, 111546.
19. Jiang, W., Bian, L., Ma, L.J., Tang, R.Z., Xun, S., and He, Y.W. (2010). Hyperthermia-induced apoptosis in Tca8113 cells is inhibited by heat shock protein 27 through blocking phospholipid scramblase 3 phosphorylation. Int. J. Hyperther. *26*, 523–537.
20. Shang, R., Lee, S., Senavirathne, G., and Lai, E.C. (2023). microRNAs in action: biogenesis, function and regulation. Nat. Rev. Genet. *24*, 816–833.
21. Peng, Y., and Croce, C.M. (2016). The role of MicroRNAs in human cancer. Signal Transduct. Targeted Ther. *1*, 15004.
22. Gandellini, P., Rancati, T., Valdagni, R., and Zaffaroni, N. (2014). miRNAs in tumor radiation response: bystanders or participants? Trends Mol. Med. *20*, 529–539.
23. Huseinovic, A., Jaspers, A., van Splunter, A.P., Sørgård, H., Wilting, S.M., Swarts, D.R.A., van der Meulen, I.H., van Beusechem, V.W., de Menezes, R.X., and Steenbergen, R.D.M. (2022). Functional Screen for microRNAs Suppressing Anchorage-Independent Growth in Human Cervical Cancer Cells. Int. J. Mol. Sci. *23*, 4791.
24. Cancer Genome Atlas Research Network (2017). Integrated genomic and molecular characterization of cervical cancer. Nature *543*, 378–384.
25. van der Zee, J. (2002). Heating the patient: a promising approach? Ann. Oncol. *13*, 1173–1184.
26. Li, Y., Tong, Y., Liu, J., and Lou, J. (2022). The Role of MicroRNA in DNA Damage Response. Front. Genet. *13*, 850038.
27. Li, J.H., Liu, S., Zhou, H., Qu, L.H., and Yang, J.H. (2014). starBase v2.0: decoding miRNA-ceRNA, miRNA-ncRNA and protein-RNA interaction networks from large-scale CLIP-Seq data. Nucleic Acids Res. *42*, D92–D97.
28. Sherman, B.T., Hao, M., Qiu, J., Jiao, X., Baseler, M.W., Lane, H.C., Imamichi, T., and Chang, W. (2022). DAVID: a web server for functional enrichment analysis and functional annotation of gene lists (2021 update). Nucleic Acids Res. *50*, W216–W221.
29. Zhang, S., Wang, W., Wu, X., Liu, W., and Ding, F. (2020). miR-16-5p modulates the radiosensitivity of cervical cancer cells via regulating coactivator-associated arginine methyltransferase 1. Pathol. Int. *70*, 12–20.

30. Du, R., Jiang, F., Yin, Y., Xu, J., Li, X., Hu, L., and Wang, X. (2021). Knockdown of lncRNA X inactive specific transcript (XIST) radiosensitizes non-small cell lung cancer (NSCLC) cells through regulation of miR-16-5p/WEE1 G2 checkpoint kinase (WEE1) axis. Int. J. Immunopathol. Pharmacol. *35*, 2058738420966087.

31. Wang, F., Mao, A., Tang, J., Zhang, Q., Yan, J., Wang, Y., Di, C., Gan, L., Sun, C., and Zhang, H. (2019). microRNA-16-5p enhances radiosensitivity through modulating Cyclin D1/E1-pRb-E2F1 pathway in prostate cancer cells. J. Cell. Physiol. *234*, 13182–13190.

32. Jin, Q., Hu, H., Yan, S., Jin, L., Pan, Y., Li, X., Peng, Y., and Cao, P. (2021). lncRNA MIR22HG-Derived miR-22-5p Enhances the Radiosensitivity of Hepatocellular Carcinoma by Increasing Histone Acetylation Through the Inhibition of HDAC2 Activity. Front. Oncol. *11*, 572585.

33. Sun, C., Shen, C., Zhang, Y., and Hu, C. (2021). LncRNA ANRIL negatively regulated chitooligosaccharide-induced radiosensitivity in colon cancer cells by sponging miR-181a-5p. Adv. Clin. Exp. Med. *30*, 55–65.

34. Ke, G., Liang, L., Yang, J.M., Huang, X., Han, D., Huang, S., Zhao, Y., Zha, R., He, X., and Wu, X. (2013). miR-181a confers resistance of cervical cancer to radiation therapy through targeting the pro-apoptotic gene. Oncogene *32*, 3019–3027.

35. Chen, G., Zhu, W., Shi, D., Lv, L., Zhang, C., Liu, P., and Hu, W. (2010). MicroRNA-181a sensitizes human malignant glioma U87MG cells to radiation by targeting Bcl-2. Oncol. Rep. *23*, 997–1003.

36. Zhu, J., Ye, Q., Chang, L., Xiong, W., He, Q., and Li, W. (2015). Upregulation of miR-195 enhances the radiosensitivity of breast cancer cells through the inhibition of BCL-2. Int. J. Clin. Exp. Med. *8*, 9142–9148.

37. Zheng, L., Chen, J., Zhou, Z., and He, Z. (2017). miR-195 enhances the radiosensitivity of colorectal cancer cells by suppressing CARM1. OncoTargets Ther. *10*, 1027–1038.

38. Tang, T., Shan, G., and Zeng, F. (2019). Knockdown of DGCR5 enhances the radiosensitivity of human laryngeal carcinoma cells via inducing miR-195. J. Cell. Physiol. *234*, 12918–12925.

39. Zhang, J., and Li, W. (2018). Long noncoding RNA CYTOR sponges miR-195 to modulate proliferation, migration, invasion and radiosensitivity in nonsmall cell lung cancer cells. Biosci. Rep. *38*, BSR20181599.

40. Cancer Genome Atlas Network (2015). Comprehensive genomic characterization of head and neck squamous cell carcinomas. Nature *517*, 576–582.

41. Song, L., Liu, S., Zeng, S., Zhang, L., and Li, X. (2015). miR-375 Modulates Radiosensitivity of HR-HPV-Positive Cervical Cancer Cells by Targeting UBE3A through the p53 Pathway. Med. Sci. Monit. *21*, 2210–2217.

42. Yang, H., Zhang, X., Zhao, Y., Sun, G., Zhang, J., Gao, Y., Liu, Q., Zhang, W., and Zhu, H. (2020). Downregulation of lncRNA XIST Represses Tumor Growth and Boosts Radiosensitivity of Neuroblastoma via Modulation of the miR-375/L1CAM Axis. Neurochem. Res. *45*, 2679–2690.

43. Jin, Y., Su, Z., Sheng, H., Li, K., Yang, B., and Li, S. (2021). Circ_0086720 knockdown strengthens the radiosensitivity of non-small cell lung cancer via mediating the miR-375/SPIN1 axis. Neoplasma *68*, 96–107.

44. Zhang, B., Li, Y., Hou, D., Shi, Q., Yang, S., and Li, Q. (2017). MicroRNA-375 Inhibits Growth and Enhances Radiosensitivity in Oral Squamous Cell Carcinoma by Targeting Insulin Like Growth Factor 1 Receptor. Cell. Physiol. Biochem. *42*, 2105–2117.

45. Wei, J., Wang, L., Sun, Y., and Bao, Y. (2020). LINC00662 contributes to the progression and the radioresistance of cervical cancer by regulating miR-497-5p and CDC25A. Cell Biochem. Funct. *38*, 1139–1151.

46. Li, J., Lei, C., Chen, B., and Zhu, Q. (2021). LncRNA FGD5-AS1 Facilitates the Radioresistance of Breast Cancer Cells by Enhancing MACC1 Expression Through Competitively Sponging miR-497-5p. Front. Oncol. *11*, 671853–672021.

47. Liu, W.H., Qiao, H.Y., Xu, J., Wang, W.Q., Wu, Y.L., and Wu, X. (2020). LINC00473 contributes to the radioresistance of esophageal squamous cell carcinoma by regulating microRNA-497-5p and cell division cycle 25A. Int. J. Mol. Med. *46*, 571–582.

48. Li, C., Fu, Y., He, Y., Huang, N., Yue, J., Miao, Y., Lv, J., Xiao, Y., Deng, R., Zhang, C., and Huang, M. (2023). Knockdown of LINC00511 enhances radiosensitivity of lung adenocarcinoma via regulating miR-497-5p/SMAD3. Cancer Biol. Ther. *24*, 2165896.

49. Nagel, R., Martens-de Kemp, S.R., Buijze, M., Jacobs, G., Braakhuis, B.J.M., and Brakenhoff, R.H. (2013). Treatment response of HPV-positive and HPV-negative head and neck squamous cell carcinoma cell lines. Oral Oncol. *49*, 560–566.

50. Buch, K., Peters, T., Nawroth, T., Sänger, M., Schmidberger, H., and Langguth, P. (2012). Determination of cell survival after irradiation via clonogenic assay versus multiple MTT Assay–a comparative study. Radiat. Oncol. *7*, 1.

51. Vandersickel, V., Slabbert, J., Thierens, H., and Vral, A. (2011). Comparison of the colony formation and crystal violet cell proliferation assays to determine cellular radiosensitivity in a repair-deficient MCF10A cell line. Radiat. Meas. *46*, 72–75.

52. Brobbey, C., Liu, L., Yin, S., and Gan, W. (2022). The Role of Protein Arginine Methyltransferases in DNA Damage Response. Int. J. Mol. Sci. *23*, 9780.

53. Huang, W., Liu, J., Hu, S., Shi, G., Yong, Z., Li, J., Qiu, J., Cao, Y., and Yuan, L. (2019). miR-181a Upregulation Promotes Radioresistance of Nasopharyngeal Carcinoma by Targeting RKIP. OncoTargets Ther. *12*, 10873–10884.

54. Bose, M.V., and Rajkumar, T. (2015). Assessment of the radiation sensitivity of cervical cancer cell lines. Methods Mol. Biol. *1249*, 351–362.

55. Roos, W.P., Thomas, A.D., and Kaina, B. (2016). DNA damage and the balance between survival and death in cancer biology. Nat. Rev. Cancer *16*, 20–33.

56. Maréchal, A., and Zou, L. (2013). DNA Damage Sensing by the ATM and ATR Kinases. Cold Spring Harbor Perspect. Biol. *5*, a012716.

57. Lord, C.J., and Ashworth, A. (2012). The DNA damage response and cancer therapy. Nature *481*, 287–294.

58. Chang, H.H.Y., Pannunzio, N.R., Adachi, N., and Lieber, M.R. (2017). Non-homologous DNA end joining and alternative pathways to double-strand break repair. Nat. Rev. Mol. Cell Biol. *18*, 495–506.

59. Takagawa, Y., Gen, Y., Muramatsu, T., Tanimoto, K., Inoue, J., Harada, H., and Inazawa, J. (2020). miR-1293, a Candidate for miRNA-Based Cancer Therapeutics, Simultaneously Targets and the DNA Repair Pathway. Mol. Ther. *28*, 1494–1505.

60. Sung, P., and Klein, H. (2006). Mechanism of homologous recombination: mediators and helicases take on regulatory functions. Nat. Rev. Mol. Cell Biol. *7*, 739–750.

61. Scully, R., Panday, A., Elango, R., and Willis, N.A. (2019). DNA double-strand break repair-pathway choice in somatic mammalian cells. Nat. Rev. Mol. Cell Biol. *20*, 698–714.

62. Xu, M., Huseinovic, A., Jaspers, A., Yuan, L., and Steenbergen, R.D.M. (2023). Downregulation of miR-193a/b-3p during HPV-induced cervical carcinogenesis contributes to anchorage-independent growth through PI3K-AKT pathway regulators. J. Med. Virol. *95*, e28589.

63. Kim, Y.K. (2023). RNA therapy. Exp. Mol. Med. *55*, 1281–1282.

64. Seyhan, A.A. (2024). Trials and Tribulations of MicroRNA Therapeutics. Int. J. Mol. Sci. *25*, 1469.

65. (2024). What will it take to get miRNA therapies to market? Nat. Biotechnol. *42*, 1623–1624.

66. Hong, D.S., Kang, Y.K., Borad, M., Sachdev, J., Ejadi, S., Lim, H.Y., Brenner, A.J., Park, K., Lee, J.L., Kim, T.Y., et al. (2020). Phase 1 study of MRX34, a liposomal miR-34a mimic, in patients with advanced solid tumours. Br. J. Cancer. *122*, 1630–1637.

67. Gomez, I.G., MacKenna, D.A., Johnson, B.G., Kaimal, V., Roach, A.M., Ren, S., Nakagawa, N., Xin, C., Newitt, R., Pandya, S., et al. (2015). Anti-microRNA-21 oligonucleotides prevent Alport nephropathy progression by stimulating metabolic pathways. J. Clin. Investig. *125*, 141–156.

68. Gebert, L.F.R., Rebhan, M.A.E., Crivelli, S.E.M., Denzler, R., Stoffel, M., and Hall, J. (2014). Miravirsen (SPC3649) can inhibit the biogenesis of miR-122. Nucleic Acids Res. *42*, 609–621.

69. Saha, I., Halder, J., Rajwar, T.K., Mahanty, R., Pradhan, D., Dash, P., Das, C., Rai, V.K., Kar, B., Ghosh, G., and Rath, G. (2024). Novel Drug Delivery Approaches for the Localized Treatment of Cervical Cancer. AAPS PharmSciTech *25*, 85.

70. Aloss, K., and Hamar, P. (2024). Augmentation of the EPR effect by mild hyperthermia to improve nanoparticle delivery to the tumor. Biochim. Biophys. Acta Rev. Cancer *1879*, 189109.

71. Ren, Y.Q., Fu, F., and Han, J. (2015). MiR-27a modulates radiosensitivity of triple-negative breast cancer (TNBC) cells by targeting CDC27. Med. Sci. Monit. *21*, 1297–1303.

72. Upraity, S., Kazi, S., Padul, V., and Shirsat, N.V. (2014). MiR-224 expression increases radiation sensitivity of glioblastoma cells. Biochem. Biophys. Res. Commun. *448*, 225–230.

73. Brady, C.S., Bartholomew, J.S., Burt, D.J., Duggan-Keen, M.F., Glenville, S., Telford, N., Little, A.M., Davidson, J.A., Jimenez, P., Ruiz-Cabello, F., et al. (2000). Multiple mechanisms underlie HLA dysregulation in cervical cancer. Tissue Antigens *55*, 401–411.

74. Lin, C.J., Grandis, J.R., Carey, T.E., Gollin, S.M., Whiteside, T.L., Koch, W.M., Ferris, R.L., and Lai, S.Y. (2007). Head and neck squamous cell carcinoma cell lines: Established models and rationale for selection. Head Neck *29*, 163–188.

75. Hermsen, M.A., Joenje, H., Arwert, F., Welters, M.J., Braakhuis, B.J., Bagnay, M., Westerveld, A., and Slater, R. (1996). Centromeric breakage as a major cause of cytogenetic abnormalities in oral squamous cell carcinoma. Genes Chromosomes Cancer *15*, 1–9.

76. Steenbergen, R.D.M., Hermsen, M.A.J.A., Walboomers, J.M.M., Joenje, H., Arwert, F., Meijer, C.J.L.M., and Snijders, P.J.F. (1995). Integrated Human Papillomavirus Type-16 and Loss of Heterozygosity at 11q22 and 18q21 in an Oral-Carcinoma and Its Derivative Cell-Line. Cancer Res. *55*, 5465–5471.

77. Snoek, B.C., Verlaat, W., Babion, I., Novianti, P.W., van de Wiel, M.A., Wilting, S.M., van Trommel, N.E., Bleeker, M.C.G., Massuger, L.F.A.G., Melchers, W.J.G., et al. (2019). Genome-wide microRNA analysis of HPV-positive self-samples yields novel triage markers for early detection of cervical cancer. Int. J. Cancer *144*, 372–379.

78. Babion, I., Miok, V., Jaspers, A., Huseinovic, A., Steenbergen, R.D.M., van Wieringen, W.N., and Wilting, S.M. (2020). Identification of Deregulated Pathways, Key Regulators, and Novel miRNA-mRNA Interactions in HPV-Mediated Transformation. Cancers *12*, 700.

79. dMIQE Group, and Huggett, J.F. (2020). The Digital MIQE Guidelines Update: Minimum Information for Publication of Quantitative Digital PCR Experiments for 2020. Clin. Chem. *66*, 1012–1029.

80. Babion, I., Snoek, B.C., van de Wiel, M.A., Wilting, S.M., and Steenbergen, R.D.M. (2017). A Strategy to Find Suitable Reference Genes for miRNA Quantitative PCR Analysis and Its Application to Cervical Specimens. J. Mol. Diagn. *19*, 625–637.

81. Livak, K.J., and Schmittgen, T.D. (2001). Analysis of relative gene expression data using real-time quantitative PCR and the 2(-Delta Delta C(T)) Method. Methods *25*, 402–408.

82. Franken, N.A.P., Rodermond, H.M., Stap, J., Haveman, J., and van Bree, C. (2006). Clonogenic assay of cells *in vitro*. Nat. Protoc. *1*, 2315–2319.

83. Brix, N., Samaga, D., Belka, C., Zitzelsberger, H., and Lauber, K. (2021). Analysis of clonogenic growth *in vitro*. Nat. Protoc. *16*, 4963–4991.

84. Chen, Y., and Wang, X. (2020). miRDB: an online database for prediction of functional microRNA targets. Nucleic Acids Res. *48*, D127–D131.

85. van de Wiel, M.A., de Menezes, R.X., Siebring-van Olst, E., and van Beusechem, V.W. (2013). Analysis of small-sample clinical genomics studies using multi-parameter shrinkage: application to high-throughput RNA interference screening. BMC Med. Genom. *6*, S1.

86. Bates, D., Mächler, M., Bolker, B.M., and Walker, S.C. (2015). Fitting Linear Mixed-Effects Models Using lme4. J. Stat. Software *67*, 1–48.

OMTN, Volume *36*

Supplemental information

High-throughput 3D spheroid screens identify microRNA sensitizers for improved thermoradiotherapy in locally advanced cancers

MengFei Xu, Mark A. van de Wiel, Dominika Martinovičová, Angelina Huseinovic, Victor W. van Beusechem, Lukas J.A. Stalpers, Arlene L. Oei, Renske D.M. Steenbergen, and Barbara C. Snoek

Table S1: Overview of miRNAs selected for the discovery screen, validation screen and subsequent functional validation, including obtained relative effect sizes. FDR, false discovery rate.

Table S2: Overview of predicted miRNA targets associated with DNA damage response, excluding targets associated with DNA repair pathways HR and NHEJ. Predicted targets for each miRNA are marked with X. miRNAs with no predicted targets— miR-27a, miR-92a, miR-106b, and miR-3158— are not included in the table.

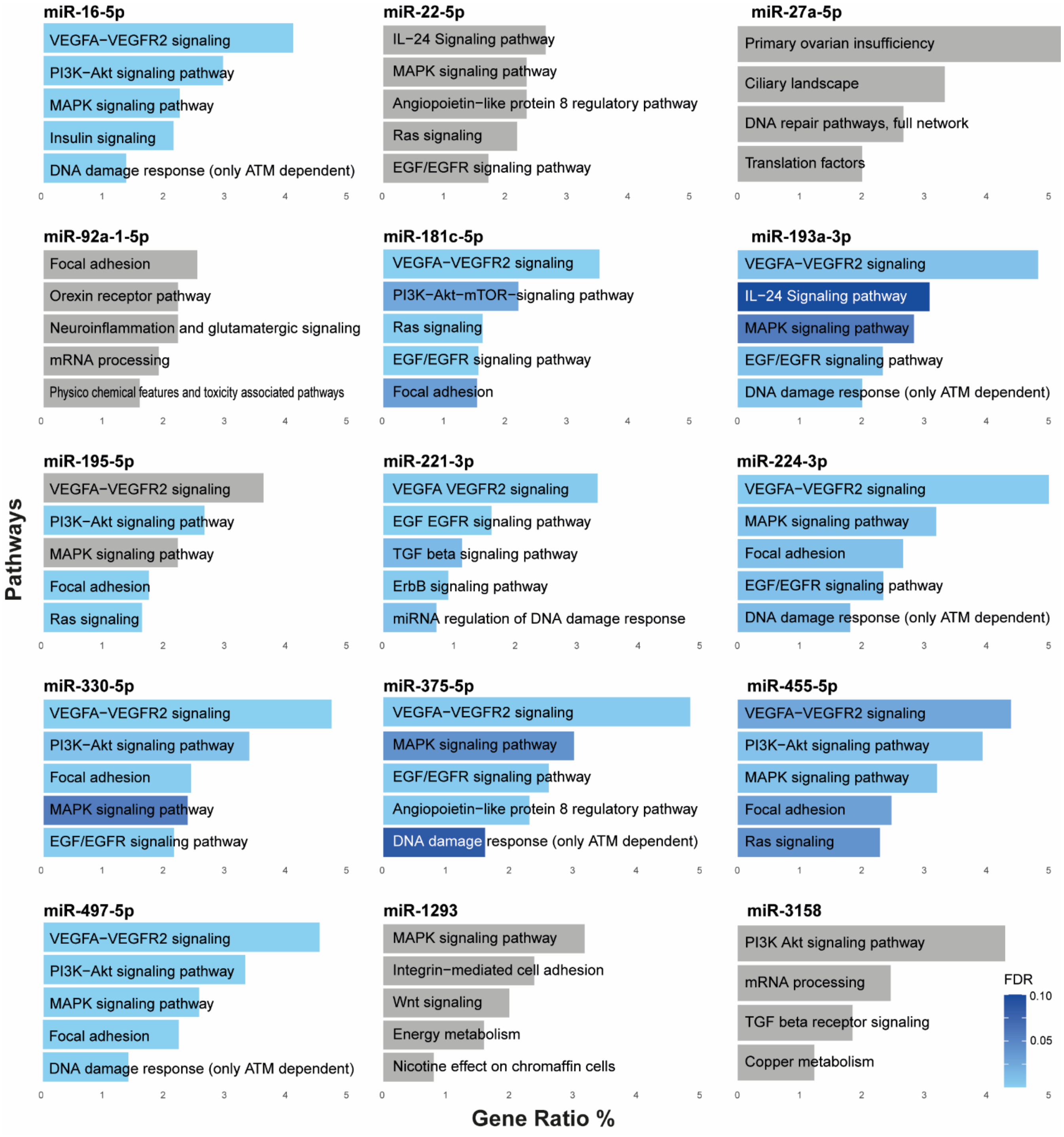

Figure S1: Overview of significantly enriched pathways for each miRNA. The top five enriched pathways or all pathways (in case less than five) were shown. Gene ratio indicates the percentage of genes over the total genes in the given pathway. Insignificant enrichments are indicated in gray. FDR, false discovery rate.

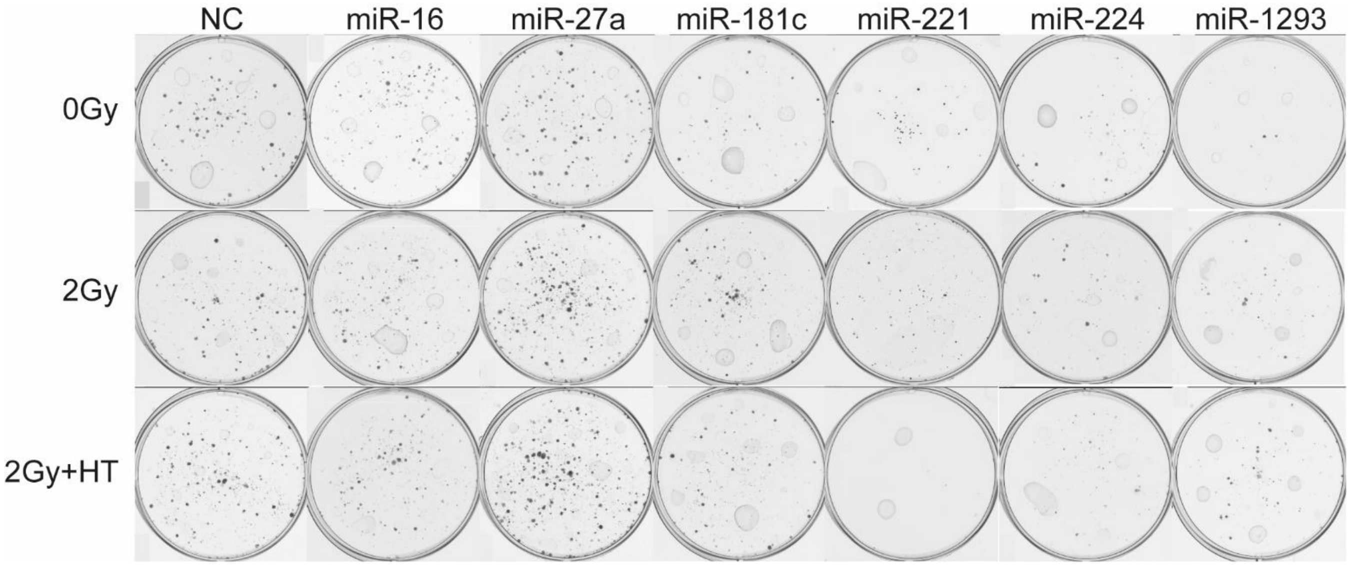

Figure S2: Clonogenic assays of different treatment conditions combined with miRNA transfection. Representative images of clonogenic assays for each miRNA are shown with non-treatment (0Gy), irradiation (2 Gy), thermoradiotherapy (2 Gy+HT) in SiHa cells.

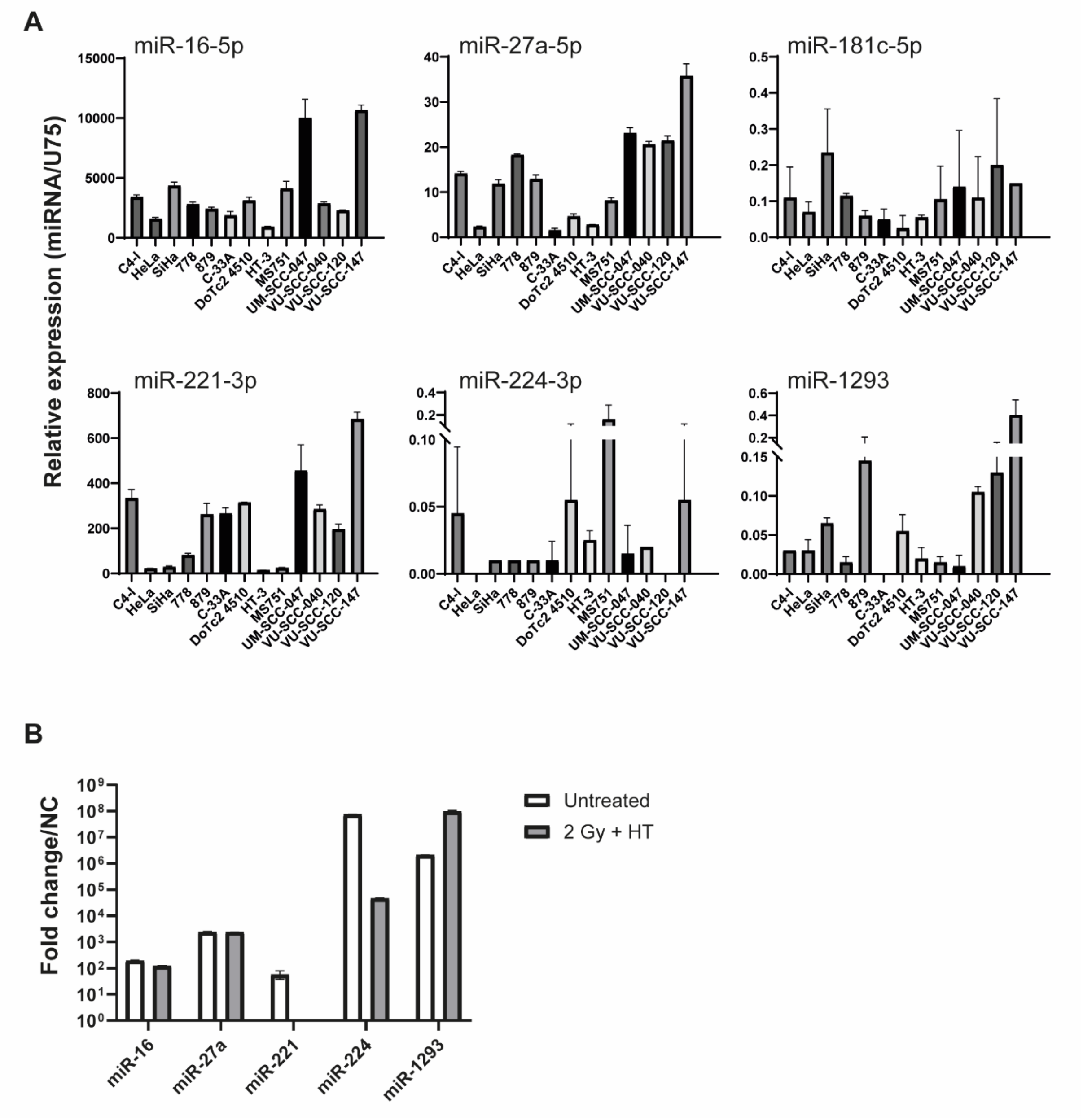

Figure S3: miRNA expression levels quantified by RT-qPCR. A: The expression level of miR-16-5p, miR-27a-5p, miR-181c-5p, miR-221-3p, miR-224-3p, and miR-1293 were quantified in relation to reference gene U75 across cancer cell lines included in the high-throughput miRNA screens. **B**: relative miRNA expression fold change compared to NC (fold change = 1) in SiHa cells 48 hours post-treatment are indicated. Untreated refers to miRNA transfected cells only, while 2 Gy+HT refers to cells that received thermoradiotherapy combined with miRNA transfection. For miR-221, only untreated cells were included. The mean ± SD are shown.

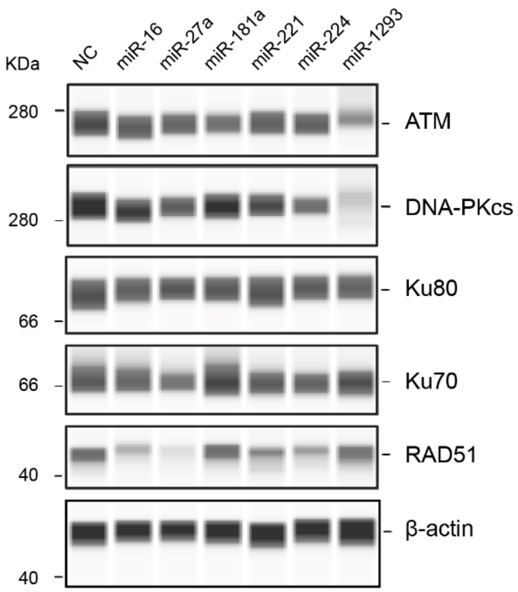

Figure S4: Western Blot analysis of predicted miRNA targets associated with DNA repair pathways. Whole cell extracts from SiHa cells collected 24 hours after miRNA mimic transfection were analyzed for ATM, DNA-PKcs, Ku80, Ku70, and RAD51 expression using Simple Western. β-actin was used as a loading control. The bands shown are digitally generated representations derived from light signal peaks of the electropherograms produced by Simple Western. Data from one of two independent experiments are shown. NC, negative control.

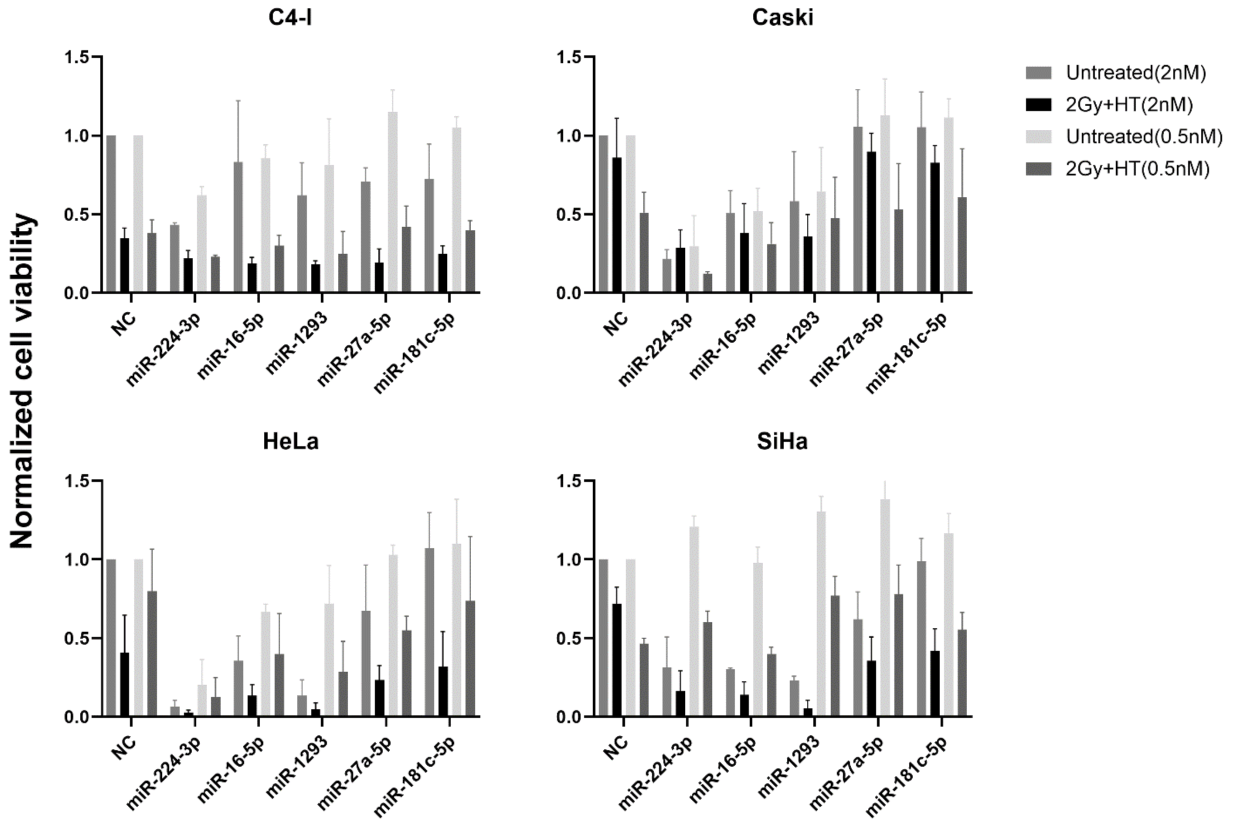

Figure S5: Cell viability results for the top five significant miRNAs from the validation screen at different miRNA mimic concentrations. Viability is normalized to the negative control (NC) for each cell line (C4-I, CaSki, HeLa, and SiHa) at each miRNA mimic concentration (2 nM or 0.5 nM). Data represent mean ± SD. Untreated refers to miRNA transfected cells only, while 2 Gy+HT refers to cells that received thermoradiotherapy combined with miRNA transfection.
